# Coordinated regulation of photosynthesis in rice increases yield and tolerance to environmental stress

**DOI:** 10.1038/ncomms6302

**Published:** 2014-10-31

**Authors:** Madana M. R. Ambavaram, Supratim Basu, Arjun Krishnan, Venkategowda Ramegowda, Utlwang Batlang, Lutfor Rahman, Niranjan Baisakh, Andy Pereira

**Affiliations:** 1 Virginia Bioinformatics Institute, Virginia Tech, Blacksburg, Virginia 24061, USA; 2 Department of Crop, Soil, and Environmental Sciences, University of Arkansas, Fayetteville, Arkansas 72701, USA; 3 School of Plant, Environmental, and Soil Sciences, Louisiana State University Agricultural Center, Baton Rouge, Louisiana 70803, USA

## Abstract

Plants capture solar energy and atmospheric carbon dioxide (CO_2_) through photosynthesis, which is the primary component of crop yield, and needs to be increased considerably to meet the growing global demand for food. Environmental stresses, which are increasing with climate change, adversely affect photosynthetic carbon metabolism (PCM) and limit yield of cereals such as rice (*Oryza sativa*) that feeds half the world. To study the regulation of photosynthesis, we developed a rice gene regulatory network and identified a transcription factor HYR (HIGHER YIELD RICE) associated with PCM, which on expression in rice enhances photosynthesis under multiple environmental conditions, determining a morpho-physiological programme leading to higher grain yield under normal, drought and high-temperature stress conditions. We show HYR is a master regulator, directly activating photosynthesis genes, cascades of transcription factors and other downstream genes involved in PCM and yield stability under drought and high-temperature environmental stress conditions.

## Abstract (web summary)

Improving photosynthetic efficiency to increase crop yield is an important goal of plant breeders. Here, Ambavaram *et al.* identify a transcription factor that is a key regulator of photosynthetic carbon metabolism in rice and show that its overexpression enhances grain yield.

## Graphical abstract

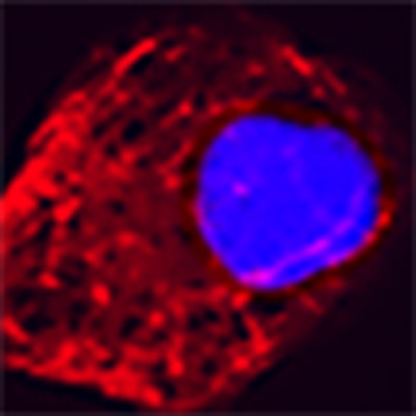

High crop yield under optimal as well as environmental stress conditions is a valuable crop-stability trait that is targeted for improvement using classical breeding as well as genetic engineering^1^. Many approaches have been proposed to boost intrinsic yield, such as enhancement of growth or increase in photosynthetic rate and capacity^2^. Photosynthesis, the basis of life on earth that converts light energy to chemical energy in integrated photosynthetic carbon metabolism (PCM) processes, is complex and requires a systems-wide approach to coordinately improve plant productivity and yield^3^ that is stable under environmental stresses. Transcription factors (TFs) have shown promise in coordinately improving specific traits in rice, such as photosynthetic assimilation and plant biomass^4^ or grain yield (GY) components under drought^5^, and have the potential to coordinately regulate photosynthesis and PCM for crop yield.

Although photosynthesis is accepted as the basis of absolute yield, yield improvement via direct improvement of photosynthetic efficiency has not yet been successful^6^. Nonetheless, evidence that elevated CO_2_ can increase leaf photosynthesis in crops by as much as 22.6% over the growing season suggests that increasing photosynthesis can increase productivity and yield^7^. One of the primary strategies has been on engineering RuBisCO to improve photosynthetic efficiency^8^, although many more metabolic reactions in PCM and associated processes in sucrose synthesis and photorespiration have been shown to play an equivalent role. Metabolic analysis using a dynamic model of PCM^9^ suggests that the partitioning of resources among enzymes of PCM in C_3_ crop leaves is not optimal for maximizing the light-saturated rate of photosynthesis, and under elevated CO_2_ predicted for the future, this problem is amplified. The selection of changes to the photosynthetic process intended to improve biomass production and crop yield must take into account a complex matrix of interacting genes and mechanisms. It is recognized that combining systems modelling with modern breeding and transgenic technologies holds promise to design new pathways, such as improved CO_2_ fixation and photorespiratory pathways^10^, or new genetic-regulatory networks^11^ to improve photosynthetic efficiency.

GY in cereals such as rice is limited by environmental stresses such as drought and high temperature, which are also increasing due to climate-change effects. Photosynthesis and related carbon metabolism is primarily affected by stress, thereby reducing GY^12^. Understanding of this complex interaction in a systems biology approach will provide the genetic tools to maintain yield under stress. Amongst cereals, rice as a paddy field crop is particularly susceptible to water stress and it is estimated that 50% of the world rice production is affected by drought. Major research efforts are directed at understanding the mechanism of plant responses to drought stress to identify gene products that confer adaptation to water deficit. Molecular mechanisms of water stress response have been investigated primarily in the model plant species Arabidopsis. Upon exposure to drought-stress conditions, many stress-related genes are induced, and their products are thought to function as cellular protectors from stress-induced damage^13^.

The expression of stress-related genes is largely regulated by TFs. The rice and Arabidopsis genomes code for >1,500 TFs, and about 45% of them are reported from plant-specific families. Various drought-stress studies have identified TF families with putative functions in drought including MYB, bZIP, Zinc finger, NAM and APETALA2 (AP2)^13^. The AP2 family is one of the plant-specific TFs whose members share a highly conserved DNA-binding domain known as AP2, and members of this family have been associated with various developmental processes and stress tolerance^14^. The AP2 TF CBF4, also known as DREB1, was shown by overexpression analysis to lead to drought adaptation in Arabidopsis^14^; the Arabidopsis AP2 TF called HARDY was reported to provide enhanced drought tolerance and water-use efficiency (WUE) in Arabidopsis and rice^4^. Ectopic expression of these genes confer drought tolerance and/or adaptation by modifying cellular structures of leaves and roots, CO_2_ exchange and parameters such as WUE, which correlate with the transformed plants’ ability to withstand drought. Taken together, these and other findings indicate that AP2 TFs offer the potential to engineer plants in a way that makes them more productive under stress conditions.

Although drought stress can alter the growth and development of a plant at any time during its life cycle, water limitations during reproductive growth stages can be especially conducive to yield losses in crops such as rice and maize (*Zea mays*)^15^. Accordingly, the reproductive phases in these plants should be an important stage to study for identifying stress-responsive genes that might have a protective, or yield-altering, function in drought. Advances in plant genomics, including the availability of the complete genome sequence of rice, have provided an opportunity to identify stress-related TFs that control yield under drought. To this end, a genome-wide analysis of drought-stress responses was conducted and led to the identification of a candidate drought-induced AP2/ERF TF in reproductive tissues.

To determine whether the TF could play a role in enhancing the tolerance of rice and possibly other crops to drought stress, transgenic plants were generated that contain the candidate gene driven by the CaMV 35S promoter. The *HYR* (*HIGHER YIELD RICE*) gene-expressing transgenic plants here are referred to as HYR lines, as they showed higher GY under well-watered and drought-stress conditions. In addition the HYR lines expressed multiple component traits involved in photosynthesis, sugar levels, root and shoot biomass and WUE under well-watered and drought-stress conditions. The enhanced productivity and the drought-resistant phenotype of the transgenic plants compared with the wild type (WT) are discussed. These studies provide an insight into improvement of plant productivity through enhancement of photosynthesis and multiple downstream biological processes (BPs) in combination with stress tolerance in plants.

## Results

### Rice regulatory association network analysis

Since environmental stresses such as drought perturb many essential BPs important for growth and development^12^, we reasoned that a global analysis of the regulation of stress responses would reveal the underlying transcription network regulating photosynthesis, PCM and growth. For PCM-related BPs—for example, photosynthesis and carbohydrate biosynthesis—we sought to identify transcription factors (TFs), whose functions were also perturbed/implicated under environmental stress. To characterize the network of genes and BPs involved in stress response and tolerance, we developed separate ‘regulatory association networks’ in rice using genome-wide expression profiles of rice genes under ‘control’ and ‘stress’ conditions (see Methods, Supplementary Fig. 1). Briefly, we used numerous publicly available whole-genome expression profiles of rice to calculate ‘specific’ correlation scores (*S*_*ij*_) of all genes in the genome to each TF, first under control conditions, and then under a variety of stress conditions. We then used these correlations to determine the association of each TF to functionally related sets of genes (diverse gene ontology (GO)^16^ BPs). The process generated two conditional—control and stress—TF-function association networks in rice. The networks individually represent the association of each of 328 ‘specific’ BP/pathway to every TF (among 3,082) in the rice genome for control and stress conditions.

**Figure 1:**
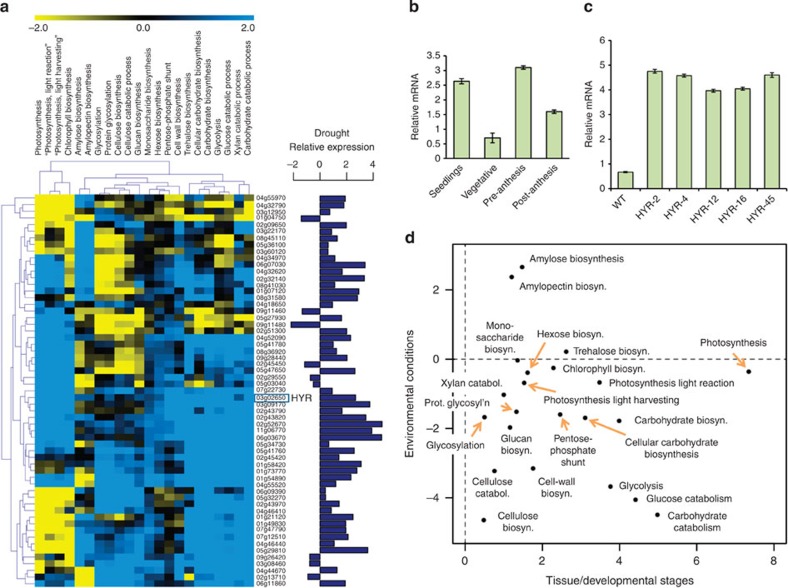
Analysis of *HYR* gene expression and association to photosynthetic carbon metabolism (PCM) processes. (**a**) Rice ‘conditional’ regulatory association network, represented by the heatmap showing specific associations of ‘carbohydrate metabolism’ (C) and ‘photosynthesis’-related (P) GO BP gene sets (along the columns) to AP2/ERF transcription factors (TFs; along the rows) under ‘control’ conditions. Blue indicates positive association and yellow indicates negative association, with *HYR* (Os03g02650) showing consistent positive association with PCM processes. The horizontal bar-plot next to the TFs represents the level of differential expression (log ratio) of these genes under drought. (**b**) Expression analysis of *HYR* in different growth stages by quantitative real-time qRT-PCR showing mean log-ratio of expression of HYR under drought compared with control, with error bars denoting the s.e.m.; *n*=3. (**c**) Expression of *HYR* in transgenic Nipponbare plants bearing the 35S-*HYR* gene shown by qRT-PCR, showing mean and s.e.m. (*n*=3). (**d**) Scatterplot showing the association scores of the PCM gene sets (‘C’ and ‘P’; Fig. 1a above) with *HYR* in two different conditional correlation networks, one built to emulate ‘control’ conditions (*x* axis) and the other to emulate ‘stress’ conditions (*y* axis). Thus, the *x*- and *y* coordinates of each point correspond to the association of a PCM gene set (for example, ‘photosynthesis’) to *HYR* under control (about 7.5) and stress (about −0.25) conditions. When the *x* and *y* values of a particular function, for example, ‘photosynthesis’, are very different, it signifies that the function’s associations with HYR is significantly altered by stress.

Using this framework, we focused on choosing candidate rice TFs that potentially play a role in growth, yield and tolerance under drought. The japonica rice genome contains 182 AP2/ERF-domain-containing TFs, many of which have been shown to exhibit functions under growth and stress^17^. We first analysed publicly available expression profiles of rice genes under drought^18^ and identified 59 drought-regulated AP2/ERF TFs (Fig. 1a). We then selected two broad (GO) functional categories, ‘carbohydrate metabolism’ and ‘photosynthesis’ (and all their descendants), related to growth and energy allocation to query the ‘control’ TF-function association network. Examination of the associations of these biological functions with the 59 AP2/ERF TFs showed that some of the AP2 TFs have large positive associations with genes annotated to these functions, and many were negatively associated with some key functional subsets. The exception was Os03g02650 (named HYR, for reasons described above); this was the only drought-inducible rice AP2/ERF TF having positive associations with all of the carbohydrate or photosynthetic functional categories. We used a similar procedure to identify HYR’s association with PCM processes in the ‘stress’ TF-functional network. Contrasting these ‘stress’ associations with the positive function associations of HYR in the ‘control’ network, we noted that most of these functions have drastically different associations with HYR (Fig. 1d). Under ‘stress’, many of the positive associations are reduced to no (zero) associations (for example, ‘photosynthesis’ and ‘chlorophyll biosynthesis’), while several others have flipped into negative associations (for example, ‘carbohydrate biosynthesis’). This analysis, thus, revealed that HYR’s positive association with PCM processes under control conditions is significantly perturbed under strong environmental stimuli (Fig. 1d), which together suggested a role for HYR in regulating PCM for basal level protection against environmental stresses.

Quantitative reverse transcriptase-PCR (qRT-PCR) experiments carried out on Nipponbare plants following 4–8 days of progressive drought, confirmed that drought causes upregulation of *HYR* in rice at different developmental stages including two critical reproductive phases—pre-anthesis (end of booting stage, panicle elongation) and post-anthesis (2 weeks after flowering) (Fig. 1b). *HYR* is predominantly induced in panicles, at about threefold at pre-anthesis and 1.5-fold at post-anthesis under severe drought relative to well-watered conditions, which include the critical reproductive phases at which drought stress reduces cereal yield^19^.

### Expression of the *HYR* gene enhances photosynthesis in rice

To assess the role of increased HYR expression in rice, an overexpression construct of the *HYR* gene under control of the CaMV 35S promoter was transformed into rice cultivar Nipponbare (see Methods for details), and five hygromycin-resistant lines (HYR-2, HYR-4, HYR-12, HYR-16 and HYR-45) were identified that showed segregation for a single hygromycin resistance locus and presence of the T-DNA locus by PCR. The five lines expressed *HYR* at approximately 2–3 fold higher than the expression level of *HYR* under drought (Fig. 1b,c).

**Figure 2:**
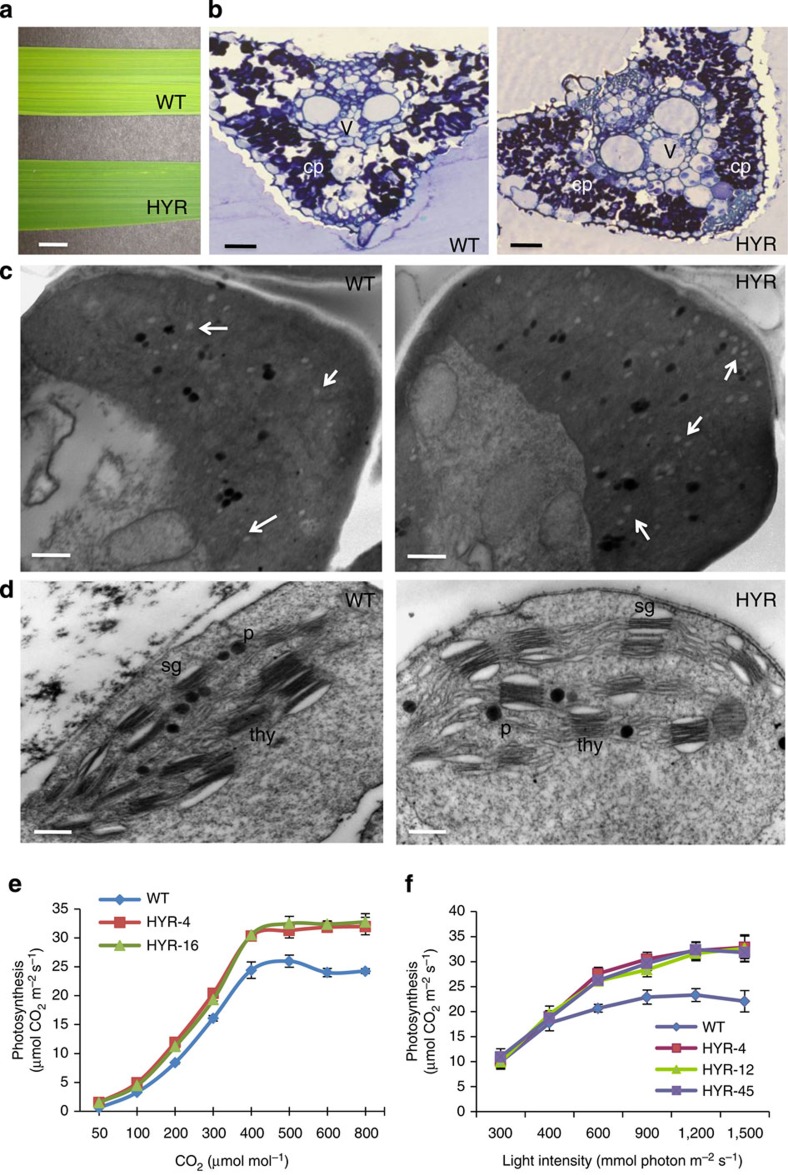
Morpho-physiological features of rice HYR lines showing enhanced photosynthesis parameters. (**a**) Leaf blade phenotype of WT (upper) and a HYR line showing the darker-green leaf surface in HYR plants; scale bar, 1 cm. (**b**) Increased number of dark-staining chloroplasts (labelled ‘cp’) in HYR compared with WT, images taken by confocal microscopy of leaf sections stained with 1% Toluidine blue and photographed ( × 40) under identical settings, vascular bundles labelled ‘v’; scale bar, 50 μm. (**c**) Flag leaf parenchyma cells of WT and HYR plants visualized by transmission electron microscopy showing increased number of white starch granules (arrows) in HYR cells; scale bar, 4 μm. (**d**) Transmission electron micrographs of WT and HYR leaves, showing thylakoid ultrastructure of mesophyll chloroplasts after drought treatment; labels are sg, starch grain; p, plastoglobulus; thy, thylakoid; scale bar, 250 nm. (**e**) Assimilation rate as a function of increasing CO_2_ concentration at saturated light intensity of 1,500 mmol m^−2^ s^−1^ in WT and HYR lines, measured by portable photosynthesis system LI-6400XT, values are means±s.e. (*n*=6). (**f**) Assimilation rate as a function of increasing light intensity at CO_2_ concentration of 370 μmol mol^−1^ in WT and HYR lines measured by LI-6400XT system, values are means±s.e. (*n*=6).

Morphological analysis showed that the HYR lines had brilliant dark-green leaves compared with the WT (Fig. 2a), with ~15% increased chlorophyll levels (Supplementary Fig. 2) and chloroplast number (Fig. 2b). HYR lines also displayed higher accumulation of starch granules in flag-leaf parenchyma (Fig. 2c), signifying a carbohydrate reserve proximal to the panicle during grain development. In response to increased CO_2_ concentration and irradiance levels HYR lines revealed increased photosynthetic capacity, as well as higher CO_2_ and light-saturation points than WT (Fig. 2e,f).

Under low irradiance (≤400 μmol photons m^−2^ s^−1^), the net CO_2_ assimilation was not much different compared with that of WT (Fig. 2f). However, at higher irradiance (400–1,500 μmol photons m^−2^ s^−1^) the HYR lines exhibited significantly higher CO_2_ assimilation compared with the WT. The increased photosynthesis of HYR lines at present CO_2_ levels of 400 p.p.m. and above (Fig. 2e) indicates a high photosynthetic capacity of the system to synthesize more carbohydrates and thereby a major component of GY.

### Rice HYR lines are drought tolerant

**Figure 3:**
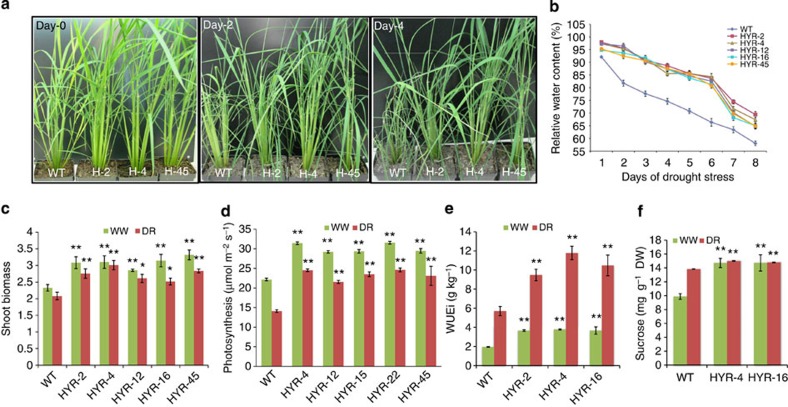
Drought physiological response phenotypes of rice HYR lines at the vegetative stage. (**a**) Effect of progressive drought (dry down) on rice WT and HYR lines at the vegetative stage: drought stress initiated at 6 weeks after germination, phenotype shown at Day 0, 2 and 4 after stress initiation. (**b**) Relative water content (RWC%) of WT and HYR lines measured at different days of stress, WT showing 75% and HYR lines maintain 85% RWC at day 4; RWC values are means±s.e. (*n*=6). (**c**–**f**) Controlled drought-stress treatment (75% field capacity) response of HYR lines, well-watered shown in green bars and drought treatments in red, values are means±s.e. (*n*=6 for **c**–**f**) and significance using *t*-test (*P≤0.05; **P≤0.01). (**c**) Comparison of shoot biomass; (**d**) gas-exchange analysis using portable photosynthesis system LI-6400XT showing photosynthetic rate; (**e**) instantaneous water-use efficiency WUEi; (**f**) total sucrose content of two HYR lines with values as mean±s.e. (*n*=4).

The five *HYR* overexpression lines were evaluated for multiple drought-tolerance physiological parameters in greenhouse tests. The HYR lines were compared with WT plants in a progressive drought experiment in which seedlings were allowed to dry down, and the HYR lines showed better growth than WT (Fig. 3a) and survived 8 days without watering. More importantly, the HYR lines maintained higher relative water content (≥65% RWC) compared with WT plants (Fig. 3b), throughout the drought treatment and indicating a physiological tolerance drought mechanism.

To make detailed drought physiological measurements at the adult plant level, the HYR and WT plants were grown side by side to the late-vegetative stage under well-watered/semi-flooded conditions for 8 weeks, and half of the plants from each genotype along with WT were allowed to dry down for 4–8 days until plants showed drought-stress symptoms but not leaf rolling. A day before gas-exchange measurements, the soil moisture in the pots with drought stress was adjusted to 75% of field capacity to maintain drought stress. The CO_2_ gas-exchange parameters showed that *HYR* lines maintained a significantly higher rate of photosynthetic carbon assimilation compared with WT under both well-watered (32% higher) and drought-stress (60% higher) conditions (Fig. 3d).

Chlorophyll fluorescence measurements of Fv/Fm (Fv is variable fluorescence and Fm maximum fluorescence) values that represent the maximum photochemical efficiency of PSII in a dark-adapted state, were 5% and 35% higher in the HYR lines, than in the WT plants under well-watered and drought-stress conditions, respectively (Supplementary Fig. 3). These results indicate that the effect of drought stress in the fluorescence parameter Fv/Fm, which is a measure of accumulated photooxidative damage to PSII, were considerably smaller in the HYR lines than in the WT plants. Transmission electron microscopy analysis of the rice genotypes under drought revealed that the structure of the chloroplasts in HYR lines under drought was not affected (Fig. 2d), whereas in WT plants their shapes changed from oblong to spherical with severely damaged thylakoid membranes under the same level of drought stress, inferring that maintenance of the HYR chloroplast membrane structure contributes to the sustained photosynthetic capacity under drought^20^.

Drought-response analysis of HYR lines was also carried out under controlled water-deficit stress in soil maintained at 75% of field capacity (see Methods). The HYR lines showed significantly higher shoot biomass under both well-watered and drought-stress conditions (Fig. 3c), contributed by the higher photosynthetic capacity (Fig. 3d), and also showed higher WUE (Fig. 3e), with no significant changes in cumulative water use and stomatal conductance (Supplementary Fig. 4). Since abscisic acid (ABA) is known to play a key role in drought-stress response of plants, HYR lines were tested for ABA response and showed less sensitivity to ABA, monitored by seed germination as well as seedling growth parameters (Supplementary Figs 5 and 6). Under drought stress, ABA mainly regulates stomatal behaviour to reduce transpirational water loss, which can reduce photosynthesis^21^.

The HYR lines and WT grown under controlled water-deficit stress were also analysed to study the effect of drought on carbohydrate metabolism. Leaf material of HYR lines and WT was harvested, and dry matter analysed for glucose, fructose and sucrose content (Fig. 3f). Drought stress increased glucose, fructose and sucrose; the HYR lines produced more free glucose, fructose, sucrose and total sugars than the WT under well-watered and drought-stress conditions (Supplementary Fig. 7). This suggests a similar hypothesis as in wheat, one of the mechanisms utilized by the plants to overcome water-stress effects via accumulation of compatible soluble sugars^22^.

### HYR promotes a vigorous root system

**Figure 4:**
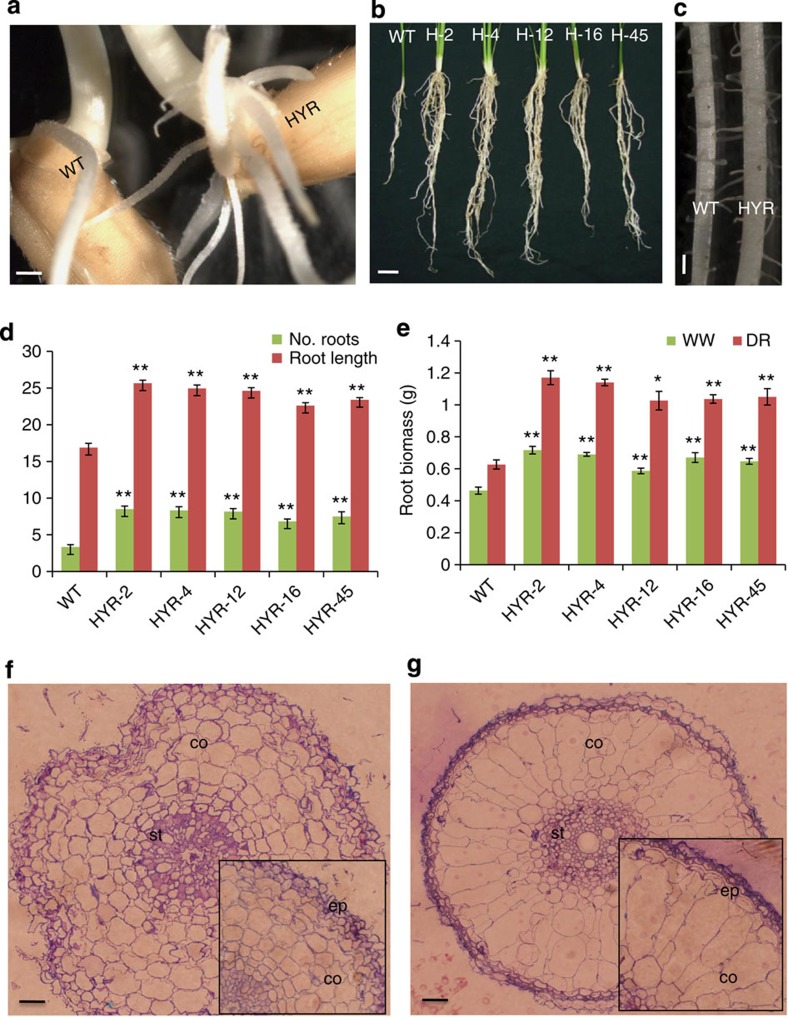
Root phenotype and response of HYR lines. (**a**) Adventitious root phenotype of HYR (HYR-4) line and WT grown on nutrient-free medium for 7 days; scale bar, 1.5 mm. (**b**) Root morphology of WT and five HYR lines grown for 30 days in sand supplemented with Hoagland’s solution; scale bar, 2 cm. (**c**) HYR line (HYR-4) showing thicker roots compared with WT, photographed under light microscope; scale bar, 2 mm. (**d**) Quantification of HYR and WT root phenotypes shown in **b**, with the number of adventitious roots and the root length (cm), represented by mean±s.e. (*n*=6). (**e**) Root biomass analysis of WT and HYR lines under well-watered and drought-stress conditions in soil. Values are means±s.e. of (*n*=6 of each genotype), with significance shown (*t*-test; *P≤0.05; **P≤0.01). (**f**,**g**) Root ultrastructure of HYR lines shown by sections (1 cm above tip) of WT (**f**) and HYR (**g**), at low ( × 20) and high ( × 40, inset) magnification under light microscope; scale bar, 150 μm. The prominent structure of enlarged cortex (co), stele (st) and epidermis (ep) are seen in HYR root sections (**g**).

Analysis of the root system of HYR lines revealed a robust root system with increased number of adventitious roots (Fig. 4a,b), which grew longer and thicker (Fig. 4b–d) than that of WT plants. Microscopic studies of the roots of HYR lines show an enlarged stele and larger size of cortical cells with expansive aerenchyma (Fig. 4f,g). Further, gravimetric analysis of root biomass in HYR lines showed a significant increase of 42% and 72% under well-watered and controlled drought, respectively, compared with the WT (Fig. 4e).

Root thickness in rice was found to confer drought resistance, as roots are capable of increasing root length density and water uptake by producing more and larger root branches^5^. The contribution of these traits clearly indicates efficient translocation and increased surface area available for the uptake of water from the soil, thus imparting drought tolerance to HYR lines. An increase in root dry weight (DW) under stress indicates remobilization of assimilates from shoot to root, and higher root biomass increases the plant’s ability to find less-available water and thus increased drought resistance. These phenotypes of increased root surface area available for water and nutrient uptake from the soil are related to field drought resistance and GY of rice^5^,^23^.

### HYR regulates GY under normal and stress conditions

**Figure 5:**
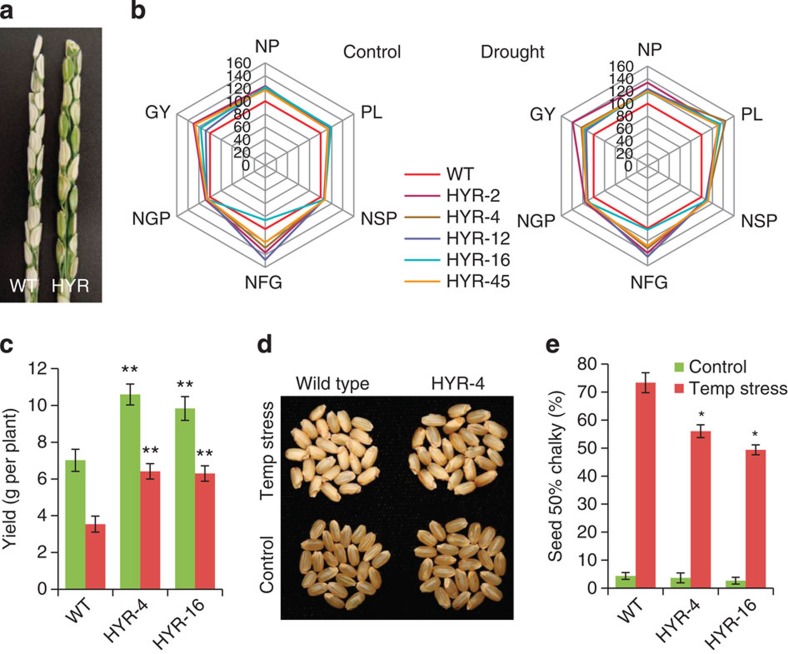
Grain yield (GY) components under normal and reproductive stage stress conditions. (**a**) Maturing spikelets of WT and a HYR line showing higher grain filling under drought stress. (**b**) GY components of HYR compared with WT grown under well-watered control and reproductive stage drought-stress conditions. In this plot each data point represents a percentage of the mean values (*n*=10), with that of WT controls set at 100% as reference. Abbreviations for the components represent: NP (number of panicles per plant), PL (panicle length), NSP (number of spikelets per panicle); NFG (number of filled grain per plant); NGP (number of grains per panicle); GY (grain yield). Values are the mean±s.e. (*n*>6) and `**' indicates significant difference from wild-type, *t*-test at *P*<0.01. (**c**) Reproductive stage high-temperature stress of WT and HYR lines, the HYR lines showing increased GY under high-temperature (red) and normal (green) conditions. (**d**) Reduced chalkiness of harvested HYR line under high nighttime temperature given during the seed development stage, signifying better grain quality. Plants at the early-boot stage were exposed to high day/night temperature of 36/26 °C until physiological maturity. (**e**) Chalkiness of HYR and WT under high nighttime temperature expressed as % of mature grain with ≥50% chalkiness. Values are the mean±s.e. (*n*>6) and ‘*’ indicates significant difference from wild type, *t*-test at *P*<0.05.

The GY potential of rice is determined by three major components: number of panicles (NPs) per plant (associated to tiller number), number of spikelets per panicle and grain weight^24^. Under well-watered conditions, the HYR lines exhibited as high as 29% increase in GY with half the genotypes ranging between 27 and 29% increase (Table 1). The increase in GY was represented in the yield components plant biomass (Fig. 3c), NP and panicle length, spikelet number per panicle (NSP) and grain number per panicle (NGP) (Fig. 5b; Table 1), corroborating other GY component studies^19^,^24^.

Drought-stress treatment at the sensitive reproductive stage showed that HYR lines produced 14–39% higher GY through improvement in multiple yield components (Figs 3c and 5a,b; Table 2), exhibiting higher biomass with larger panicles and more grains than WT under drought conditions (Fig. 5b). This means that GY increased due to higher single-grain weight and grain number and NSP in HYR. In summary, the results indicate that the HYR lines produced larger panicles with more and larger grains, as well as more total biomass compared with WT under well-watered and even drought-stress conditions. An increase in the number of filled grains might be due to the contribution of carbohydrates from photosynthesis, with more and efficient translocation into the grain and thus increase in the GY^24^. The above results clearly indicate that HYR plays a significant role in conferring drought tolerance and improving GY under drought in rice.

Heat stress is another problem affecting rice yield worldwide due to increases in nighttime temperature caused by climate change^25^. The response of HYR lines to high day and night temperatures was tested at flowering and grain maturation. The HYR lines showed increased GY under high temperatures (Fig. 5c), independent of spikelet sterility and grain weight (Supplementary Fig. 8), and more fertilized spikelets developed to maturity. Because high nighttime temperatures decrease grain quality^26^, the harvested threshed grain were examined for chalkiness, which showed significant reduction in HYR lines (Fig. 5d,e), supporting a mechanism of HYR maintaining photosynthate supply and starch deposition in the developing grain under high nighttime temperature.

### HYR regulates expression of genes in PCM and stress response

To identify genes and BPs regulated by HYR, gene expression profiles of WT and a HYR line were analysed by microarrays and a set of genes confirmed by qRT-PCR (Supplementary Fig. 9). GO analysis revealed that photosynthesis and PCM processes were upregulated by HYR (Supplementary Table 1; Supplementary Data1; Supplementary Fig. 10), and corroborates the positive association of HYR with PCM in the TF-function association network (Fig. 1a). These results indicate that HYR is a key regulator of genes involved in PCM processes essential for plant growth and yield (Fig. 1d), and its upregulation confers drought and heat tolerance.

**Figure 6:**
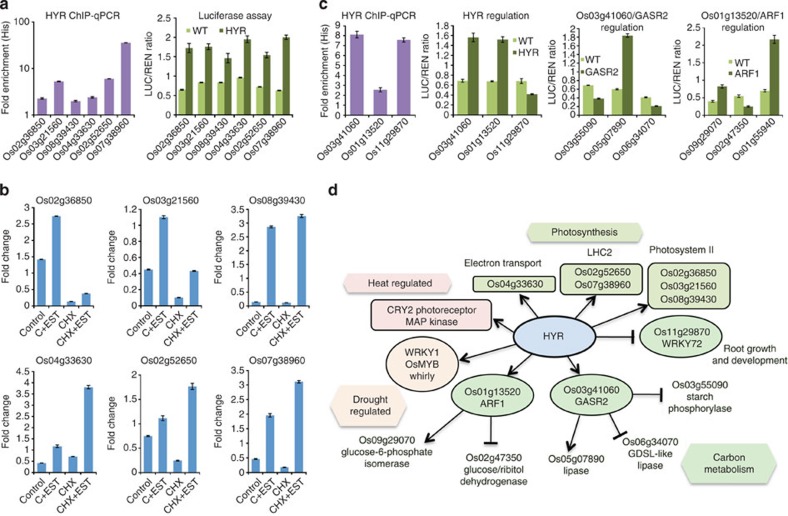
HYR is a transcriptional regulator of photosynthesis and related morpho-physiological processes. Three experimental methods (see Methods) to describe HYR function are denoted in graphs with different colours: ChIP-qPCR (purple) of HYR TAP-tagged plants assayed for HYR protein binding to promoters *in planta* (comparing, dual firefly-Renilla (LUC/REN) luciferase transactivation assays (light/dark green) with effector (TF expression) and reporter (promoter) constructs co-transformed in rice protoplasts, and steroid estradiol (EST)-inducible estrogen receptor (HER) assays (blue) with cycloheximide (CHX) treatment to prove direct transactivation of target gene promoter–reporter constructs (see Methods). All data presented are means of three biological replicates tested for significance using the *t*-test (*P*≤0.01), with significant treatments shown compared with relevant controls. (**a**,**b**) HYR binds to photosynthesis gene promoters and directly activates their transcription; the genes are involved in PSII (Os02g36850, Os03g21560 and Os08g39430), electron transport (Os04g33630) and LHC2 (Os02g52650 and Os07g38960). (**c**) HYR directly transcriptionally activates the PCM-regulating TFs GASR2 and ARF1, and represses OsWRKY72 shown by ChIP-qPCR and luciferase transactivation. Expression of the TFs GASR2 and ARF1 in luciferase transactivation assays show regulation of downstream genes (activation/repression), additional evidence direct regulation using HER fusions in EST/CHX assays (see Methods). (**d**) Model of HYR transcriptional regulatory network with direct transactivation shown by lines ending in arrows or repression by lines ending in bars. Gene directly regulated by HYR are shown as ovals (TFs) or rounded rectangles (other genes), and indirectly regulated by HYR are shown open. Hexagons designate pathways/processes regulated by HYR with the individual genes or functions highlighted in same colour as function names.

To obtain evidence of the HYR regulatory network, rice transformants expressing affinity-tagged (tandem affinity protein (TAP)-tagged) HYR protein were used to isolate HYR-bound chromatin^27^, and assayed by chromatin immunoprecipitation (ChIP)-qPCR for binding to promoters of predicted HYR-regulated genes. Promoter sequences of photosynthesis genes, PCM TFs and drought- and temperature-regulated genes were found enriched in the chromatin complex by qPCR analysis (Fig. 6a,c; Supplementary Figs 11–13), demonstrating binding of the HYR protein to promoters of these genes *in vivo*. Transcriptional regulation of HYR target photosynthesis genes (Fig. 6a) and PCM TFs (*GASR2*, *ARF1* and *WRKY72*) (Fig. 6c) was confirmed by transactivation assays in rice protoplast transformation experiments expressing *HYR* co-transformed with target promoter-luciferase constructs. The PCM genes predicted to be downstream targets of HYR-regulated TFs (GASR2/ARF1) revealed transcriptional regulation by GASR2/ARF1 in co-transformation experiments (Fig. 6c). Similarly, drought-regulated and heat-regulated genes predicted to be targets of HYR were also shown to be regulated by HYR in promoter-luciferase transactivation assays (Supplementary Figs 12,13).

The rice *WRKY72* gene, orthologue of the Arabidopsis *WRKY75* gene that induces root growth when repressed and involved in phosphate acquisition^28^, is interestingly also repressed by *HYR* expression (Supplementary Fig. 11). This was further tested for regulation of the expansin gene EXPA8 (Supplementary Fig. 14), identified from gene expression data of HYR lines, which had been shown to improve root growth and architecture^29^. Overexpression of *HYR* in rice protoplasts represses WRKY72 and induces EXPA8, while overexpression of WRKY72 represses EXPA8. This supports the hypothesis that WRKY72 regulates the expression of EXPA8, which is known to affect rice root growth and architecture^29^, and provides an explanation for the enhanced root growth in HYR lines.

To confirm direct regulation of target genes, human oestrogen receptor (HER) protein fusions^30^ of HYR and downstream TFs (*GASR2* and *ARF1*) were tested in estradiol induction assays with cycloheximide that inhibits new protein synthesis (Fig. 6; Supplementary Figs 11–13). These assays demonstrated that HYR is a direct transcriptional activator of the photosynthesis and heat-responsive genes identified, and of other co-regulated TFs that in turn transcriptionally activate or repress genes involved in carbon metabolism and drought response (Fig. 6).

The broader significance of HYR regulation of plant processes was explored further. Since HYR increases GY through a regulatory network of genes, we asked the question whether genes known to be involved in different yield components were regulated by HYR. A set of genes (*RCN*, *MOC1*, *TB1*, *LAX* and *GIF*) involved in different components of yield^31^ were tested for their expression in HYR lines and showed differential expression. There was enhanced expression of tillering genes *MOC1* and *TB1*, and panicle differentiation gene *RCN*, and repression of panicle development gene *LAX* and grain-filling gene *GIF* (Supplementary Fig. 15). To assess the potential role of HYR in diverse rice germplasm, the expression of *HYR* in rice plants was tested by qPCR and showed significant differences in expression between genotypes under normal growth, with higher expression of *HYR* in genotypes N22, Vandana and Pokkali (Supplementary Fig. 16) that are known to express traits for GY under stress^32^.

## Discussion

Improvement of GY is the primary objective in breeding and improvement of cereal crops. Rice lines overexpressing the *HYR* gene produce up to 29% increase in GY grown under well-watered ambient conditions (Fig. 5b; Table 1), showing a general improvement in all yield components in most HYR lines. The HYR lines showed increased levels of net photosynthesis measured under ambient conditions, as well as with increasing levels of CO_2_ and light intensity. This enhanced photosynthetic capacity is supported by an increase in chloroplast number and chlorophyll content, and also represented by increase in the photosynthetic products of soluble sugars, starch and plant biomass. These results concur with previous studies that have shown increased biomass production is concomitant with improved photosynthesis in Arabidopsis^10^ and rice^4^.

As a result of increased photosynthesis, total soluble sugars including sucrose, the primary phloem-mobile carbohydrate, are increased in HYR lines (Fig. 3f; Supplementary Fig. 7), but do not seem to cause the expected feedback inhibition of photosynthesis^33^. This could be due to the putative feedback loop (whereby ‘excess’ sucrose inhibits photosynthesis) that is less sensitive in HYR lines, or the sucrose levels at critical sites (presumably the chloroplasts) are less because of stronger sink activity in the larger, faster-growing plants. The evidence suggests that the higher sink capacity of growing roots, flag leaf, shoots and developing grain of HYR plants can be a reservoir for the increased photosynthate, reducing the sugar accumulation in leaves and the downregulation of photosynthesis^34^.

Under progressive drought stress with the soil allowed to dry down, HYR lines survived longer than WT controls (Fig. 3a). The higher RWC observed in the HYR lines under drought could be due to the accumulation or presence of sugars, especially sucrose, leading to osmotic adjustment. In osmotic adjustment, leaves develop a more negative osmotic potential by accumulating solutes. They can then maintain a higher RWC during a period of leaf water-potential reduction. Solute accumulation and osmotic adjustment have been associated with drought tolerance in many crop plants^12^. With controlled drought-stress treatment (see Methods), the rice HYR lines maintained higher photosynthesis and WUE, resulting in higher shoot and root biomass compared with WT plants. Since the WUE of HYR lines increased, although the cumulative water use and transpiration remained the same as WT, the enhanced photosynthetic CO_2_ assimilation determines the improved productivity of HYR lines. In C_3_ plants, WUE is generally determined by, among other factors, stomatal control of the ratio of the instantaneous rates of photosynthesis and transpiration. Stomatal closure typically leads to decrease in photosynthetic CO_2_ assimilation due to restricted diffusion of CO_2_ into the leaf and altered CO_2_ metabolism that is mainly responsible for the decline in photosynthesis in C_3_ plants under drought^35^. However, the HYR lines have reduced sensitivity to ABA (Supplementary Figs 5 and 6) and therefore have lower stomatal response to drought stress with less effect on stomatal conductance. The results for HYR plants imply that the WUE is not determined by stomatal control of photosynthesis, but by effective CO_2_ fixation as reported previously for another C_3_ plant^36^, suggesting that CO_2_ metabolism of HYR lines is more resistant to dehydration.

Soluble carbohydrates (glucose, sucrose, fructose, sorbitol and mannitol) have been reported to accumulate in plants under drought stress^37^. This is due to a shift in C-partitioning from non-soluble carbohydrates (starch) to soluble carbohydrates, which can help maintain turgor for a longer period during drought and also participate in stress-protective functions^37^. Under drought stress the accumulation of soluble carbohydrates can maintain plant turgor and contribute to stress-protective functions, such as maintenance of RWC in HYR lines due to osmotic adjustment, a mechanism of drought tolerance^38^.

The increase in number of filled grains, evident in HYR lines under drought or high-temperature stress, can be due to efficient translocation of carbohydrates from photosynthesis into the grain and increase in GY as suggested previously^39^. Silencing studies of the rice *OsBP-73* gene that reduced photosynthetic rate, NGP and filled spikelets^20^, indicate an association between these processes. Likewise, even small increases in the rate of net photosynthesis were shown to cause large increases in biomass and yield in wheat^40^. The results described here characterize the role of the TF HYR in coordinating the expression of genes involved in enhancement of the energy source photosynthesis and PCM, along with an increase of biomass and GY sink, which are sustained under normal and environmental stress conditions.

GY from rice plants is severely affected by reduction in the NPs and/or spikelets, when they are exposed to drought stress at the reproductive stage^13^. If the drought is severe during the grain-filling period, grain filling can be impaired and mean grain weight reduced. In rice, grain-filling process depends on two main carbon resources: photosynthetic assimilates and carbohydrates stored during pre-anthesis and transported to the grain from vegetative tissues. Under drought stress at pre-anthesis stage, the number of spikelets and total GY declined markedly. Moreover tiller (panicle) number per plant was also reduced in WT and some extent in HYR lines. Competition for assimilates during stem extension is believed to be an important factor influencing tiller and spikelet mortality under drought^13^. However, the lower reduction in the filling rate and number of spikelets of HYR plants under drought conditions implies that the developmental processes for panicles and spikelets had been protected from drought stress, indicating drought tolerance at the reproductive stage. HYR lines showed about 14–40% more yield under drought stress compared with the WT, probably due to the contribution of carbohydrates because of higher photosynthetic rate, which are efficiently translocated into the panicle and thus increase GY. Even small increases in the rate of net photosynthesis can translate into large increases in biomass and hence yield^40^. These studies along with our observations in HYR, deduce a positive correlation between leaf photosynthesis and crop biomass and/or yield, evidence of the essential relationship between photosynthesis and crop yield.

The rice genome is predicted to contain 139 AP2/ERF-domain-containing TF genes^17^. Other AP2 TFs have been previously found to provide enhanced root strength and increased number of secondary and tertiary roots in transgenic Arabidopsis and rice plants^4^,^5^. HYR is one of the novel AP2/ERF TFs that impacts multiple processes by regulation of biochemical pathways, growth, development and response to drought and heat. This work provides a functional characterization of the gene for its effect in plant productivity under well-watered and water-limiting conditions.

Functional analysis of the HYR protein activity as a TF show that HYR is primarily involved in direct transcriptional activation of multiple photosynthesis-related processes (Fig. 6d), HYR is also involved in a regulatory cascade activating the auxin-responsive TF *ARF1* involved in vegetative growth and seed development in rice^23^,^41^, capable of binding to the *DRO1* drought avoidance gene promoter^23^. HYR was shown to repress *OsWRKY72*, which is the orthologue of Arabidopsis *AtWRKY75* that induces root growth when repressed^28^, supporting the HYR-enhanced root growth phenotype. This was experimentally verified by expressing HYR and *OsWRKY72* in rice protoplasts, HYR expression causing WRKY72 repression and EXP8 induction, and WRKY72 overexpression causing EXP8 repression (Supplementary Fig. 14)

HYR induces transcription of the glucose-6-phosphate isomerase gene. This glycolytic enzyme PGI is localized to plastids and is required for starch accumulation in Arabidopsis^42^. Through transcriptional activation of the gibberellin-mediated regulation of *GASR2* TF, HYR also causes repression of starch phosphorylase (*OsPHO1*) responsible for reversible phosphorylation of starch precursors and starch accumulation^43^. These and other carbon metabolism genes support the role of HYR in coordinate regulation of PCM genes for biomass accumulation. These genes are coordinated in expression with yield-related genes involved in tillering, *MOC1* and *TB1*, and the panicle differentiation gene *RCN*^31^. The HYR-mediated regulation of genes that are induced by heat and drought implies a role for HYR in the stress-response regulatory pathway leading to tolerance and robust plant growth. One of genes regulated by heat and HYR, *CRY2*, is a blue-light photoreceptor^44^ involved in regulation of leaf sheath elongation, and thereby growth and biomass.

The above results provide strong evidence that HYR is a master regulator of multiple BPs, directly acting as an activator/repressor of TFs and other genes in a network involved in PCM and stress-protective pathways (Fig. 6; Supplementary Figs 11–13). The morpho-physiological programme regulated by HYR expression conditioning superior photosynthetic capacity under elevated CO_2_, light and temperature offers the potential use of HYR-expressing plants to maintain crop growth and yield under environmental stresses associated with climate change.

## Methods

### Rice regulatory association network analysis

To identify transcriptional regulators (TFs), of PCM-related BPs (photosynthesis and carbohydrate biosynthesis), whose functions were perturbed/implicated under environmental stress, we developed a ‘regulatory association network’ in rice as follows (Supplementary Fig. 1). Genome-wide expression profiles of 35,161 rice genes (denoted here without the locus loc_ prefix identifier) from various tissue/developmental stages under control conditions (243 samples; 6 data sets) were aggregated in the form of a normalized gene expression matrix (*E*_*ij*_) (Supplementary Table 2). Raw data were downloaded from Gene Expression Omnibus and background corrected, quantile normalized and summarized using robust multi-array average (RMA)^45^ based on a custom computable document format (CDF)^46^ and replicate values were averaged. The distribution of gene expression values within a developmental stage were then scaled to have mean 0 and s.d. 1, resulting in the expression matrix *E*. Next, 3,100 rice TFs were curated from multiple public databases^16^,^47^, out of which 3,082 were present in the rice Affymetrix microarray. Using the expression matrix *E*, Pearson correlations were calculated between all gene pairs^48^, which were then Fisher Z-transformed^49^ and standardized to get normalized correlations with a *N* (0,1) distribution. From these values, correlations between gene-TF pairs (35,161times;3,082) were extracted to populate a gene-TF matrix *C* with each entry *C*_*ij*_ corresponding to the normalized correlation of gene *i* with TF *j*. Following the CLR algorithm^50^, for each correlation value *C*_*ij*_ between TF *j* and a potential target gene *i*, its likelihood given the background distribution of correlations for all TF-gene pairs that involve either TF *j* or gene *i* were computed. This was done by combining the two *z*-scores of *C*_*ij*_ compared with the distribution of *C*_*i*_ and *C*_*j*_ values using Stouffer’s method^51^ to acquire ‘specific’ correlation scores (*S*_*ij*_) of all genes in the genome to each TF. Using functional annotations from GO (see below), these gene-level correlation scores were summarized into more robust process-level association scores to the TF^52^, to generate a ‘control’ TF-process association network in rice. We repeated the same procedure with a set of rice expression profiles under a variety of stress conditions (~130 samples; 10 data sets) aggregated in the form of a normalized gene expression matrix (*E*_*ij*_) (Supplementary Table 2). Calculating process/function-level association scores to TFs using this expression data resulted in a ‘stress’ TF-process association network in rice.

### Functional annotations of rice genes

Functional information—GO BP annotations—for rice genes was obtained from AgBase^53^. A gene annotated to a term was also annotated to its ancestors in the GO hierarchy following the ‘is_a’ and ‘part_of’ relationships. Then, to retain only ‘specific’ annotations, terms that annotate <1,500 genes were selected. Furthermore, to remove high redundancy in these annotations—large overlaps between genes annotated to several GO terms—going down the list of terms sorted based on the number of annotated genes, for each term, any term lower in the list that satisfied the following two conditions were removed: (i) differed in the number of genes by <5, and (ii) shared a number of genes with a Jaccard coefficient of 0.9. This provided a set of non-redundant specific GO BPs for enrichment analysis and in building the TF-process association network.

### Vector construction and rice transformation

The rice *HYR* gene (Loc_Os03g02650) as annotated in the database of rice TFs^47^, was used to make an overexpression construct by assembling the individual fragments (CaMV 35S promoter, *HYR* gene Os03g02650 and CaMV 35S terminator) with appropriate compatible cohesive ends and ligated into the binary vector pCAMBIA1301 as described below. The CaMV 35S promoter fragment from −526 to the transcription start site was obtained as a 0.55-kb HindIII–SalI fragment from a pBS-SK+ derivative of pDH51 (ref. 54). The full-length coding region of rice *HYR* was amplified using *pfu* DNA polymerase from genomic DNA of rice cv. Nipponbare, and a CaMV 35S terminator fragment was obtained as a 0.21-kb PstI–EcoRI fragment from a pBS-SK+ derivative of pDH51 (ref. 54). The construct was made in the binary vector pCAMBIA1301 containing a CaMV 35S-hygromycin phosphotransferase-tNos for selection during transformation.

A TAP-tagged *HYR* construct was made in pUC19 assembling the fragments of the CaMV 35S promoter^54^ and the NOS terminator, coding sequence for six His repeat (6 × His), a 9 × myc peptide, a 3C protease cleavage site and two copies of the IgG-binding domain (2 × IgG-BD) together known as the TAP tag^55^. The entire cassette was cloned between the Xba1–EcoR1 sites of pMOG22 (Zeneca-Mogen), which contains a chimeric CaMV 35S-hygromycin phosphotransferase-tNos for selection during transformation.

Agrobacterium-mediated transformation of *Oryza sativa* ssp. *japonica* cv. Nipponbare, plant regeneration and selection conditions were performed^56^, using the Agrobacterium strain LBA4404. Regenerated transgenic plantlets were transferred to environmentally controlled growth chambers maintained at 28 °C±1 day and 25 °C±1 night temperature, 65% relative humidity (RH) with a daily photoperiodic cycle of 14 h light and 10 h dark, and the plants were grown in soil till maturity under greenhouse conditions.

### Chlorophyll fluorescence and gas-exchange parameters

Chlorophyll fluorescence was measured using the modulated chlorophyll fluorometer OS1-FL (Opti-Sciences Inc, USA). Flag leaves of stressed and unstressed genotypes was placed in close contact with the photosynthetically active radiation clip, which provides basic data to the OS1-FL system on ambient conditions. The maximal quantum yield of PSII was calculated as Fv/Fm=(Fm−Fo)/Fm, where the minimum fluorescence (Fo) was recorded after dark adaptation for 10 min and the maximum fluorescence (Fm) was monitored by application of a 0.8-s saturating light pulse (6,000 μmol photons m^−2^ s^−1^) from white LED light. Gas-exchange measurements were done using a LI-6400XT (LI-COR Inc., NE, USA) in attached leaves of WT and *HYR* transgenic plants under well-watered and drought-stress conditions. CO_2_ gas-exchange measurements were performed after 4 h of illumination with a daily photoperiodic cycle of 14 h light and 10 h dark at leaf temperature of 25 °C, CO_2_ at 400 μmol s^−1^ and RH of 55–60%. Instantaneous WUE (WUEi) was calculated as described^4^ using the formula: WUEi=(Pn/*E*).

### Measurements of chlorophyll and RWC

Chlorophyll was extracted from 2-week-grown seedlings with 80% acetone, and determined as described^57^. Plant water status was determined by measurement of RWC (%)^58^, in which the leaf used for photosynthesis measurement was excised, and an ~6-cm section had its fresh weight (FW) determined immediately. The leaf sections were floated in deionized water at room temperature for 6 h, their rehydrated weight (RW) determined, dried in an oven at 70 °C overnight and weighed to obtain the DW. The RWC% was calculated as: RWC%=(FW−DW)/(RW−DW) × 100.

### Drought-stress treatment and WUE analysis

Controlled drought treatment was done on rice lines at late-vegetative developmental stages grown in 250-ml pots filled with a 1:1 mix of top-soil and compost, and placed in water-filled trays to simulate flooded/paddy conditions, supplemented with a general-purpose 20-20-20 fertilizer dissolved in water to provide 50 kg N, P_2_O_5_ and K_2_O ha^−1^ applied weekly during the growing period. Twenty-eight days after planting (DAP), the pots were adjusted to equal weights (soil+water) by adding water as needed, mulched with a layer of perlite of fixed weight to minimize evaporative water loss and placed on tared bases. Multiple pots of each genotype were divided in two sets, for drought-stress treatment and well-watered controls. In the drought-stress treatments pots were dried down to ~70% field capacity. At this time (31 DAP), shoots on half of the plants (four for drought and four for control treatment) for each line were harvested and dried at 72 °C for 96 h and this biomass was designated as BIO-31. For the remaining plants, gravimetric soil moisture was maintained at 70% (drought stress) or at field capacity (well-watered) by replacing water lost through transpiration. The amount of water added daily for each pot was noted for calculation of cumulative water used (WUc) for a watering schedule of 14 days. At that point (45 DAP), shoots were harvested and dried at 72 °C for 96 h, and this biomass was designated BIO-45. WUc was calculated using the formulae: daily WU=(Total weight)−(tared pot+tared base+tared mulch), and WUc=∑(daily WU) over 14 days. Gravimetric WUE (WUEg) was calculated as: WUEg=[(BIO-45)−(BIO-31)]/(WUc).

### Measurement of sugar composition and content

For sugar analysis^59^, plants were harvested (above-ground biomass) and dried at 40 °C for 72 h. Sugars were extracted from 20-mg ground samples in 2 ml of 80% ethanol in an 80 °C water bath for 15 min. The cooled crude extract was centrifuged at 3,000*g* for 10 min, 20 mg charcoal added to the supernatant, the extract centrifuged at 2,200*g* for 15 min, 20 μl transferred to a microtitre plate and dried at 50 °C for 1.5 h. A series of standard solutions of glucose, fructose and sucrose were co-analysed with the extracts. After drying, 20 μl per well deionized-distilled water was added, and after 1 h, 100 μl of glucose reagent (Sigma, St Louis, MO); the plate was kept at room temperature for 30 min, and glucose measured on a microplate reader (SpectroMax plus 386, Molecular Devices Corp., Sunnyvale, CA) at 340 nm. Ten μl of 0.25 enzyme unit (EU) phosphoglucose isomerase was next added to each well, incubated at room temperature for 30 min and fructose measured at 340 nm. Ten μl of 83 EU invertase solution was next added and incubated for 30 min before measuring at 340 nm for sucrose.

### Reproductive stage drought stress and GY analysis

For drought treatment at the reproductive stage, plants were grown in 500-ml pots in environmentally controlled growth chambers (14 h light/10 h dark cycles with light intensity 600 μmol m^−2^ s^−1^ and around 65% RH), fertilized regularly with a general-purpose fertilizer (N:P:K 20-20-20) and maintained well-watered conditions until the panicle-emergence stage^60^. Drought treatments were started at the pre-anthesis stage by withholding water for 4–8 days followed by re-watering, and physiological state monitored by chlorophyll fluorescence and gas-exchange measurements. The plants that survived after re-watering and growth till maturity (>95%), and well-watered controls were maintained under well-watered condition, with minimal six plants per genotype per treatment for GY component analysis.

At maturity/stage R9 (ref. 60), the panicle on the main culm was harvested, and spikelets with grains and unfilled spikelets were counted. The grains (caryopses) with hulls (palea and lemma attached) were threshed by hand and dried at 37 °C for 7 days and subsequently weighed. The main culm was also harvested and dried at 70 °C for 72 h and weighed. The yield components assessed were NPs per plant, panicle length, NSP, number of filled grains per plant, spikelet fertility (number of spikelets with filled grains divided by the total number of spikelets), NGP, GY (weight of grain per plant) and average single-grain weight (GY divided by grain number). The harvest index was calculated as the ratio of total grain weight to total above-ground DW.

### High-temperature stress treatment

For high-temperature stress during the vegetative stage, 50-day-old plants were transferred into a controlled environmental growth chamber (Conviron Model PGW36, Winnipeg, Manitoba, Canada) set at day/night temperature of 34/24 °C for 10 days, with light intensity 60 cm above the canopy 600 μmol m^−2^ s^−1^ and RH of 65%. For reproductive stage temperature stress, plants at the early-booting stage were exposed to high day/night temperature of 36/26 °C for 20 days. The panicles affected by high temperature were marked and used for analysis of yield components. At both vegetative and reproductive stage, a set of control plants were maintained in the greenhouse conditions with day/night temperature of 26/22 °C, light intensity of 800 μmol m^−2^ s^−1^ and 65% RH. The day/night cycles for temperature and photoperiod were 10 h day and 14 h night in both growth chamber and greenhouse.

### Histochemical staining and microscopy

One-week-old hydroponically grown roots of HYR and WT plants were excised, fixed and stained with 0.5% (w/v) uranyl acetate at 4 °C overnight as described^5^. Samples were dehydrated through an ethanol gradient, embedded in Spurr’s medium and 1-μm ultrathin sections made with a diamond knife by an ultra-microtome (RMC MTXL). The stained sections were examined and photographed with a light microscope (Nikon Eclipse E600). For transmission electron microscopy, samples were embedded in LR White resin, thin sections (50 nm) made with an ultra-microtome (RMC MTXL) and collected on nickel grids. The sections were stained with uranyl acetate and lead citrate and viewed with a JEM-1010 electron microscope (JEOL) operating at 60 kV.

### Gene expression analysis

For Affymetrix GeneChip analysis, total RNA was isolated from leaf tissue of 1-month-old HYR lines (HYR-4, HYR-12 and HYR-16) along with WT plants plant tissue using the RNeasy plant kit (Qiagen, USA). The RNA quantity/quality was measured using the Agilent 2100 Bioanalyzer (Agilent Technolgies, USA), and 4 μg of total RNA was used to generate first-strand complementary DNA with a T7-Oligo(dT) primer. Following second-strand synthesis, *in vitro* transcription was performed using the GeneChip IVT Labelling Kit. The preparation and processing of labelled and fragmented complementary RNA targets, as well as hybridization to rice Affymetrix GeneChips, washing, staining and scanning were carried out according to the manufacturer’s instructions ( http://www.affymetrix.com).

For qRT-PCR analysis three independent biological replicates were used with protocol following the comparative threshold cycle (Ct) method of quantitation with the actin or ubiquitin gene as reference^61^. For each sample, 2 μg total DNAse-treated RNA was used with GoScript Reverse Transcription System (Promega), qRT-PCR experiments were carried out using GoTaq qPCR Master Mix (Promega) with ubiquitin as standard in a CFX-96 Bio-Rad thermocycler (Bio-Rad). Melting-curve analysis was done by applying increasing temperature from 55 to 95 °C (0.5 °C/10 s), and gel electrophoresis of the final product confirmed single amplicons. Untranscribed RNA was also run as a negative control to check DNA contamination. The relative difference in expression for each sample in individual experiments was determined by normalizing the Ct value for each gene against the Ct value of ubiquitin or actin and was calculated relative to a calibrator using the equation 2^−ΔΔCt^ (ref. 62).

### Analysis of differential gene expression

Raw data from the HYR expression microarray experiment were background corrected, normalized and summarized according to the custom CDF^46^ using RMA^45^, followed by nonspecific filtering of genes that do not have enough variation (interquartile range across samples<median interquartile range) to allow reliable detection of differential expression. A linear model was then used to detect differential expression of the remaining genes^63^. The *P* values from the moderated *t*-tests were converted to *q*-values to correct for multiple hypothesis testing^64^, and genes with *q*-value <0.01 were declared as differentially expressed in response to HYR expression (compared with WT).

Genes differentially expressed in response to HYR were tested for enrichment of specific GO BPs using the hypergeometric test. To adjust for multiple comparisons, a Benjamini–Hochberg false discovery rate^65^ (*q*-value) was calculated from the *P* values, and a *q*-value threshold of 0.01 was used for significance. The results from the enrichment analysis were visualized in the form of a gene set graph, with significantly overlapping gene sets connected by an edge and the network visualized using Cytoscape^66^.

### HYR ChIP and regulation of genes

HYR:TAP-tagged chromatin was used for ChIP assays^67^. Rice leaf samples (5 g) were crosslinked in buffer containing formaldehyde (2%), nuclei isolated and the chromatin sheared by sonication into 200–600-bp-sized fragments^68^. Sonicated chromatin was precleared with 100 μl protein A agarose for 1.5 h, and immunoprecipitated into three fractions: with anti-His (R932-25, Invitrogen, 1.5–2 μg) and anti-MYC (AHO0062, Invitrogen, 1.5–2 μg) for HYR-bound chromatin, and anti-Histone H3 (AHO1432, Invitrogen, 1.5–2 μg) conjugate as nonspecific chromatin. Chromatin-bound DNA fragments were eluted after reverse crosslinking by incubation overnight with proteinase K at 37 °C, repeated again at 65 °C overnight and DNA purified following the manufacturer’s instructions. Real-time PCR was carried out in a CFX-96 thermal cycler (Bio-Rad) using the qPCR master mix (Promega) with the cycle 95 °C for 3 min, 40 cycles of 95 °C for 15 s and 59 °C for 1 min. Fold enrichment for DNA bound to chromatin isolated with the specific anti-His antibody to the nonspecific anti-Histone H3 was calculated using the formula: Ct (target)−Ct (nonspecific Ab)=dCt (ref. 27), normalized against the calibrator using the equation 2^−ΔΔCt^ (ref. 62). The ChIP experiments were performed with three biological replicates, qPCR assay done in triplicate with primers from putative HYR-regulated gene promoters for each ChIP assay (Supplementary Table 3), of differentially expressed genes based on HYR microarray data and tested for significance using the *t*-test (*P*≤0.01).

To obtain purified HYR protein for electrophoretic mobility shift assay (EMSA) experiments, a full-length HYR fragment was amplified using primers containing attB1 and attB2 sites, respectively, and cloned into pDEST42 (C-6 × -His tag Gateway expression vector) (12276-010, Invitrogen) vector using the Gateway cloning strategy (BP and LR reaction system, Invitrogen) and transformed into *Escherichia coli* strain BL-21. The bacterial protein (6 × -His-tagged HYR) was induced with 1 mM isopropyl-β-D-thiogalactoside and purified using Ni-NTA agarose (Invitrogen) following the manufacturer’s instructions. Specific sets of primers (250 bp) spanning the binding sites (GCC core) were used to amplify the promoter region of target genes of HYR genomic DNA of Nipponbare rice as template (Supplementary Table 4). Gel-purified promoter fragments were labelled using the Biotin 3′ End DNA Labelling Kit (Pierce). The protein–DNA-binding assay, according to the manufacturer’s instructions (Light Shift Chemiluminescent EMSA kit), was carried out in binding buffer (10 mM Tris–HCl, pH 7.5, 50 mM KCl, 1 mM dithiothreitol), by incubation on ice for 10 min after adding 100-fold excess of unlabelled competitor DNA (gel-purified promoter DNA fragments) followed by the addition of labelled DNA and further incubation on ice for 20 min before loading on to a 5% native polyacrylamide gel. The resolved DNA–protein complexes were electroblotted onto Nylon membrane (Biodyne) and gel shift was detected using Chemidoc (Bio-Rad) as described^68^.

For transactivation dual luciferase assays of HYR and other TFs, constructs with: (a) putative promoter targets amplified using primers (Supplementary Table 5) and cloned upstream of the firefly luciferase, (b) a CaMV 35S Renilla luciferase construct control for transformation and (c) the coding regions of HYR or other TF genes (ARF1, WRKY72 and GASR2) cloned in pUC19 between the CaMV 35S promoter and NOS terminator, were co-transformed into rice protoplasts. Luminescence was measured by the Glomax Luminometer (Promega) and the relative luciferase activity calculated^68^. The data presented are means of three biological replicates and tested for significance using the *t*-test (*P*≤0.01).

To prove direct activation of promoters by a TF, an oestrogen-inducible expression system (HER)^30^ was developed in rice, ‘HER’ constructs of HYR and its target downstream genes (ARF1, GASR2 and WRKY72) were generated by ligating the PCR-amplified complementary DNAs (primers shown in Supplementary Table 5) at the *Kpn*1 sites fused with the regulatory regions of HER at the C terminus^30^ between the CaMV 35S promoter and NOS terminator in pUC19. These constructs were used for analysis of direct transactivation of promoters in rice.

### Statistical analysis

The Student’s *t*-test was used for statistical analysis of the data in the experiments of gas-exchange measurements, chlorophyll fluorescence, gravimetric WUE, biomass measurements, root length, soluble sugars and qRT-PCR. GY components were subjected to analysis of variance and the means were tested using Fisher’s least significant difference test using statistical analysis software ( www.sas.com). In all the experiments *t-*tests performed were two sided, and quantitative differences between the two groups (for example, WT and HYR lines) of data for comparison were deemed statistically significant at *P*≤0.01 or *P*≤0.05 as indicated for each comparison in the figures and tables.

## Author contributions

M.M.R.A. performed the plant phenotypic, physiological, molecular and biochemical experiments. S.B. performed ChIP, protein-interaction and transactivation experiments. A.K. conducted the bioinformatics and statistical analysis on gene expression and network analysis. V.R. performed temperature stress and photosynthesis experiments. U.B. generated the rice transgenic plants and conducted plant phenotypic and physiological experiments. L.R. generated and analysed transgenic rice lines. N.B. supported the generation of transgenic lines and stress tests. A.P. designed and supervised the research, and together with M.M.R.A., A.K., V.R. and S.B. analysed data and wrote the manuscript.

## Additional information

**Accession codes**: Microarray data are deposited in the NCBI Gene Expression Omnibus (GEO) under accession number GSE60936.

**How to cite this article:** Ambavaram, M. M. R. *et al.* Coordinated regulation of photosynthesis in rice increases yield and tolerance to environmental stress. *Nat. Commun.* 5:5302 doi: 10.1038/ncomms6302 (2014).

## Supplementary Material

Supplementary Information Supplementary Figures 1-16, Supplementary Tables 1-5.

Supplementary Data Set GO Biological processes enrichment analysis of rice HYR lines

## Figures and Tables

**Table 1:** Grain yield component traits of HYR lines grown under well-watered conditions.

**Genotype**	**No. of panicles per plant (NP)**	**Panicle length (PL)**	**No. of spikelets per panicle (NSP)**	**No. of filled grains per plant (NFG)**	**No. of grains per panicle (NGP)**	**Grain yield (g) (GY)**	**% Increase GY**
WT	9.2^a^	19.5^a^	72.9^a^	223.9^a,b^	76.4^a^	13.8^a^	
HYR-2	11.4^b^	22.05^b^	77.3^b^	306.4^c,d^	82.5^b^	17.9^b^	29.7
HYR-4	11^b^	23.04^b^	77.7^b^	289.8^c,d^	80.1^ab^	17.53^b^	27
HYR-12	11.3^b^	22.82^b^	77.8^b^	331.9^d^	80.5^b^	14.85^c^	7.6
HYR-16	11.2^b^	23.15^b^	78.3^b^	193.3^a^	81.2^b^	16.08^d^	16.5
HYR-45	10.8^b^	22.09^b^	78.6^b^	270.7^b,c^	80.6^b^	17.52^b^	26.9
LSD 0.05	1.53	2.43	4.15	54.71	3.72	0.79	
*P* value	0.056	0.040	0.082	<0.0001	0.046	<0.0001	

LSD, Fisher’s least significant difference test.

Means within columns followed by different letters^a–d^ are significantly different at *P*≤0.05 (*n*=10), *P* values were determined by the LSD test.

**Table 2:** Grain-yield component traits of HYR lines grown under drought-stress conditions.

**Genotype**	**No. of panicles per plant (NP)**	**Panicle length (PL)**	**No. of spikelets per panicle (NSP)**	**No. of filled grains per plant (NFG)**	**No. of grains per panicle (NGP)**	**Grain yield (g) (GY)**	**% Increase GY**
WT	8^a^	15.26^a^	67.4^a^	194.2^a^	68.4^a^	9.96^a^	
HYR-2	10.7^b^	20.02^b^	72.6^a^	271.1^b^	79.4^b^	13.86^b^	39.1
HYR-4	9.6^c^	21.99^c^	74.5^b^	256.9^c^	76.6^bc^	12.22^c^	22.7
HYR-12	9.9^bc^	20.33^b^	73.3^b^	282.7^b^	76.1^c^	11.35^d^	13.9
HYR-16	9.5^c^	20.56^bc^	74.2^b^	199.1^a^	77.9^bc^	11.76^cd^	18
HYR-45	9.6^c^	19.91^b^	75.6^b^	249.8^c^	77.5^bc^	11.94^cd^	19.8
LSD 0.05	0.99	1.46	3.05	20.96	2.88	0.81	
*P* value	<0.0001	<0.0001	<0.0001	<0.0001	<0.0001	<0.0001	

LSD, Fisher’s least significant difference test.

Means within columns followed by different letters^a–d^ are significantly different at *P*≤0.05 (*n*=10), *P* values were determined by the LSD test
