# Supplementary material for: Coordinated regulation of photosynthesis in rice increases yield and tolerance to environmental stress
Source: Nat Commun. 2014 Oct 31;5:5302. doi: 10.1038/ncomms6302 (PMC4220491; doi:10.1038/ncomms6302)
Supplement: Supplementary Information — Supplementary Figures 1-16, Supplementary Tables 1-5. [file ncomms6302-s1.pdf]

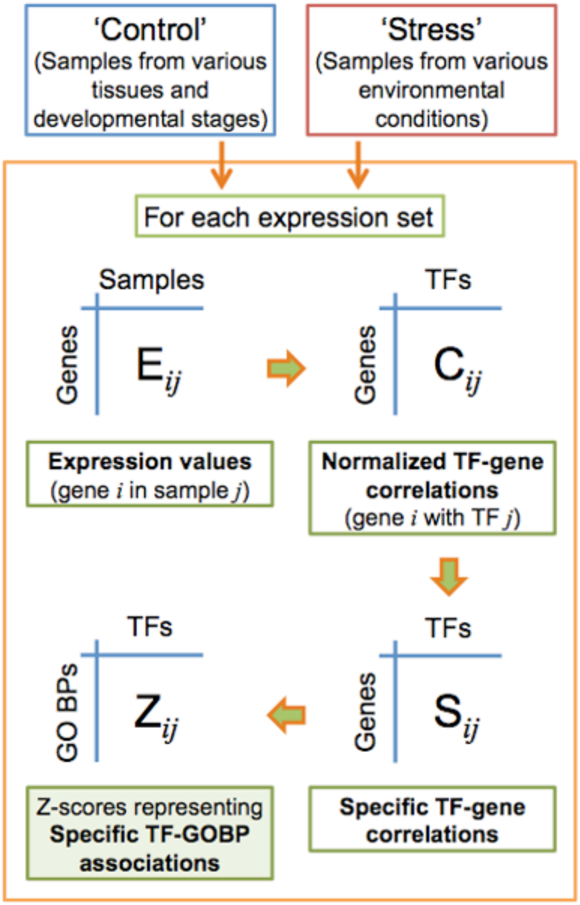

Supplementary Figure 1. Development of a rice regulatory association network.

Genome-wide expression profiles of rice genes from various developmental stages and from environmental stress conditions were used to create separate normalized gene expression matrices (E_{ij}) as described in Online Methods. Pearson correlations were calculated between all gene pairs in the expression matrix E, which were then Fisher Z-transformed to get normalized correlations with a N (0,1) distribution. These values were used to extract correlations between 3082 transcription factors and 35,161 rice genes, to generate a gene-TF matrix C_{ij} representing the normalized correlations of gene *i* with TF *j*, which was further used to derive specific TF-gene correlation scores (S_{ij}). The gene-level correlation scores were further summarized with functional annotations from GO to derive a TF-process association network in rice for prediction of TFs regulating specific biological processes.

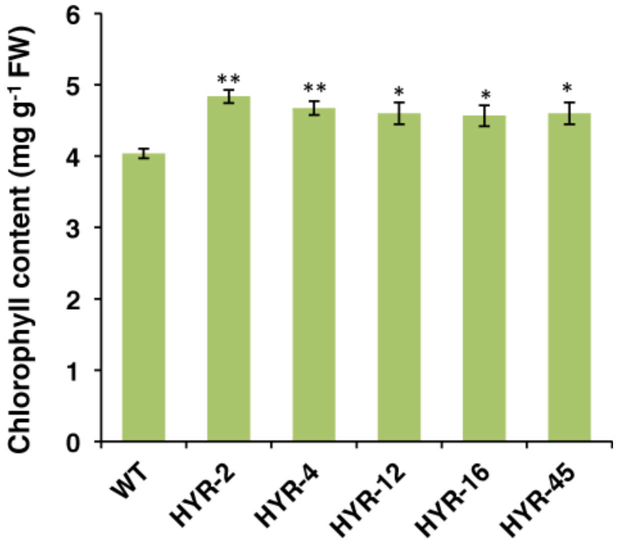

Supplementary Figure 2. Chlorophyll content of WT and HYR lines shown in **Fig. 2a**. Bars represents mean ± s.e.m. (n=6), with significance shown (t-test; *, P ≤0.05; **, P ≤0.01). Chlorophyll was extracted from 2-week grown seedlings, and quantified by absorbance measured spectrophotometrically at 645 and 663 nm.

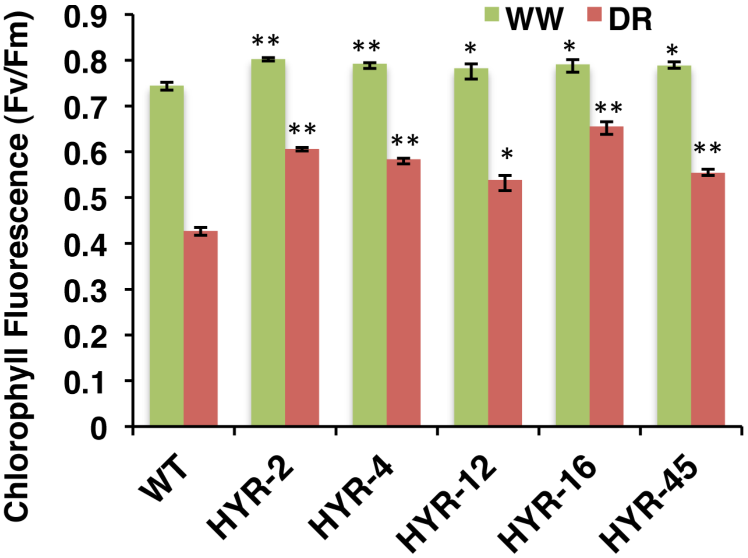

Supplementary Figure 3. Chlorophyll fluorescence parameter Fv/Fm measured using OS1-FL Chlorophyll Fluorometer. Bars represents mean ± s.e.m. (n=6), with significance shown (t-test; *, P ≤0.05; **, P ≤0.01).

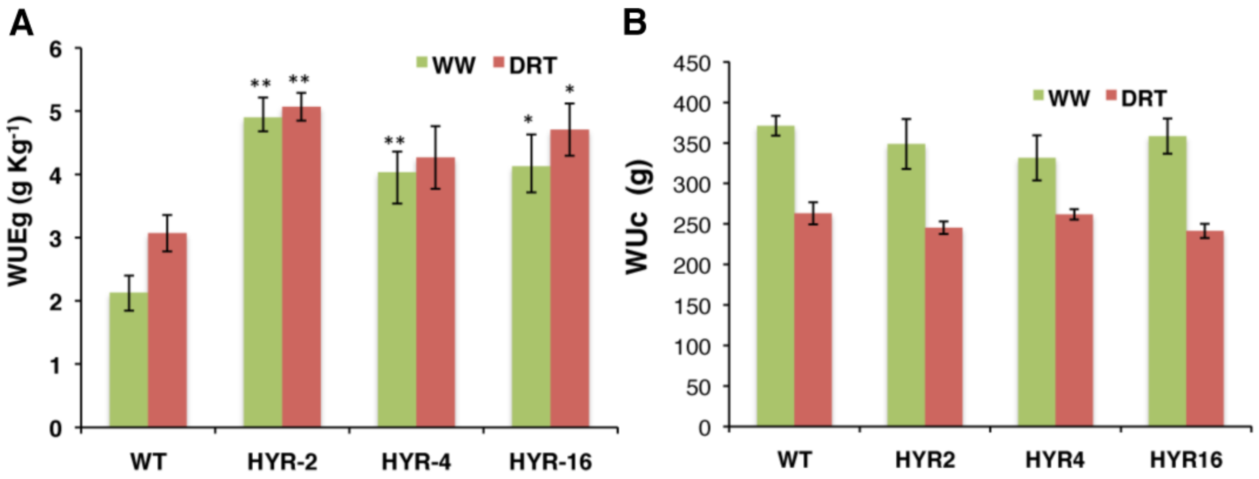

Supplementary Figure 4. Gravimetric water use efficiency (WUEg) and cumulative water use (WUc) of WT and HYR rice lines under well-watered (WW) and controlled drought (DRT) conditions of experiment shown in **Fig. 3c,d.** This supports that the higher WUE observed with no significant change in WUc is not due to reduction in stomatal transpiration but due to increase in plant biomass. Bars represents mean ± s.e.m. (n=6), with significance tested (t-test; *, P ≤0.05; **, P ≤0.01), showing non-significant difference.

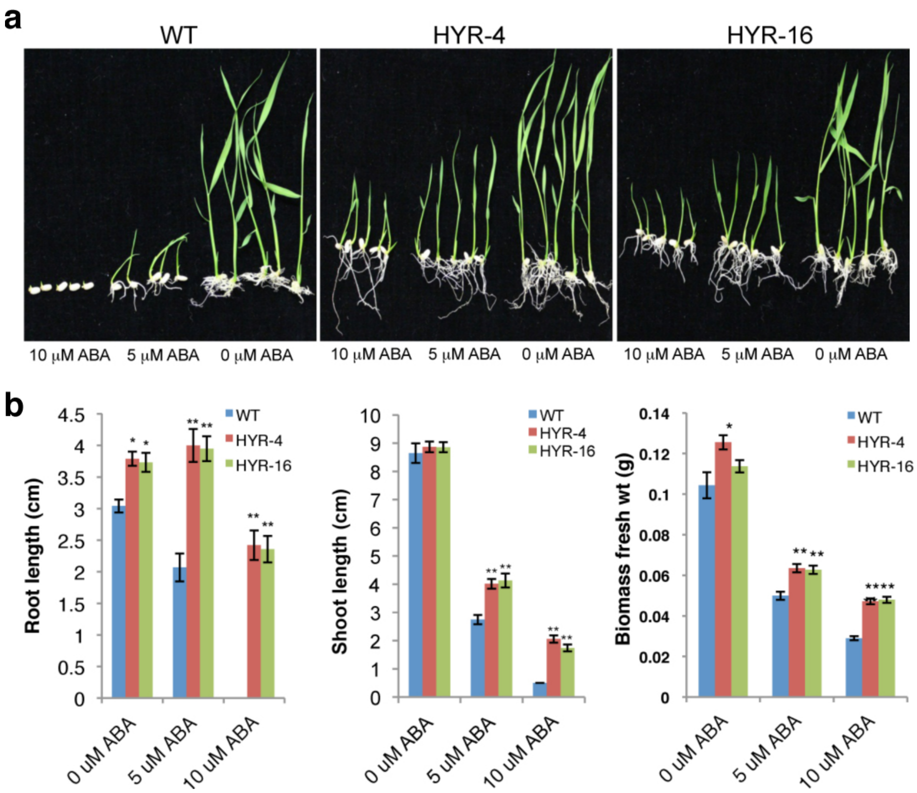

Supplementary Figure 5. Germination of wild-type and HYR lines on different concentrations of ABA. The de-hulled rice seeds were surface sterilized and transferred to MS medium supplemented with different concentrations of ABA. (**a**) Wild-type (WT) and HYR lines germinated on different concentrations of ABA for 5 days. (**b**) Germination percentage of WT and HYR seeds under different concentrations of ABA for 5 days. Values are the mean ± s.e.m. (n ≥ 30).

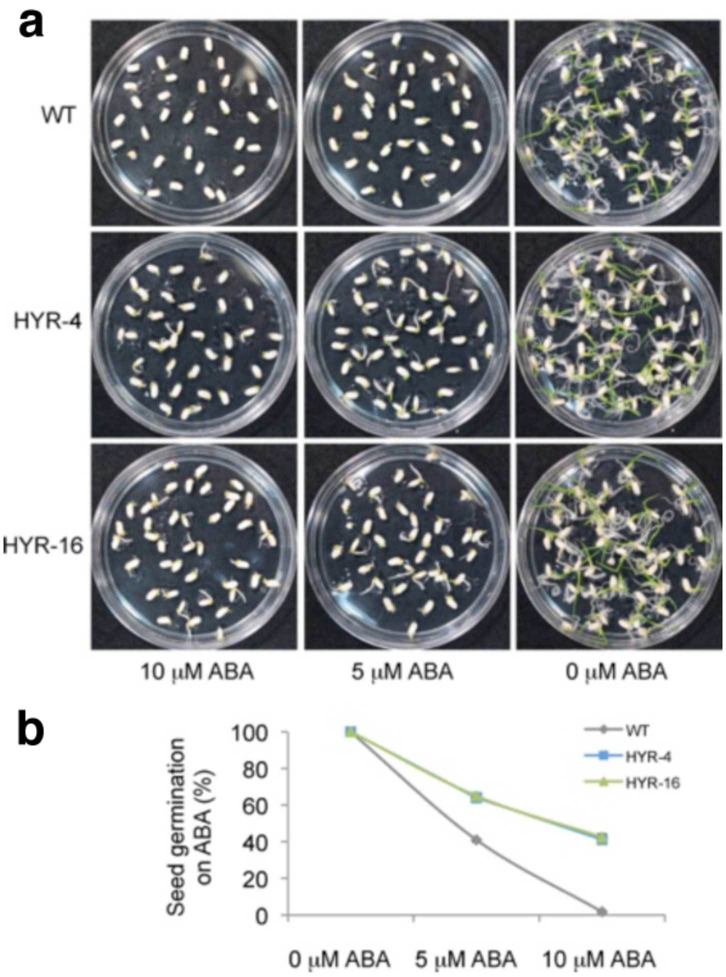

Supplementary Figure 6. Growth performance of HYR lines on different concentrations of ABA. The de-hulled seeds were surface sterilized and pre-germinated on Whatman filter paper for 2 days. Uniformly growing pre-germinated seeds were transferred on to MS medium containing different concentrations of ABA. (**a**) Plant growth and (**b**) growth performance parameters of wild-type and HYR seedlings grown on different concentrations of ABA for 7 days. Values are the mean ± s.e.m. (n ≥ 19); * and ** indicate significant difference from wild-type, t-test at P≤ 0.05 and P≤ 0.01, respectively.

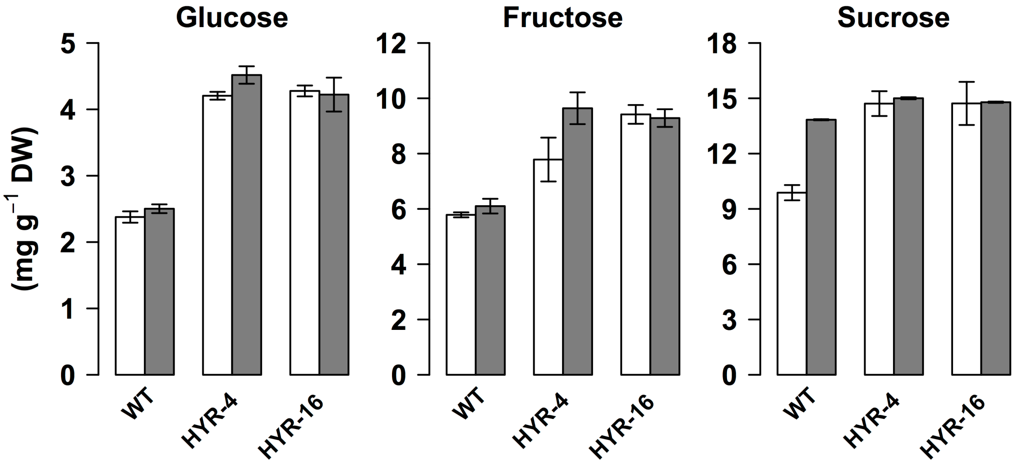

Supplementary Figure 7. Analysis of soluble sugars (glucose, fructose and sucrose) in WT and HYR lines under well watered (white bars) and drought stressed (grey bars) conditions. Error bars show mean ± s.e.m. (n=6).

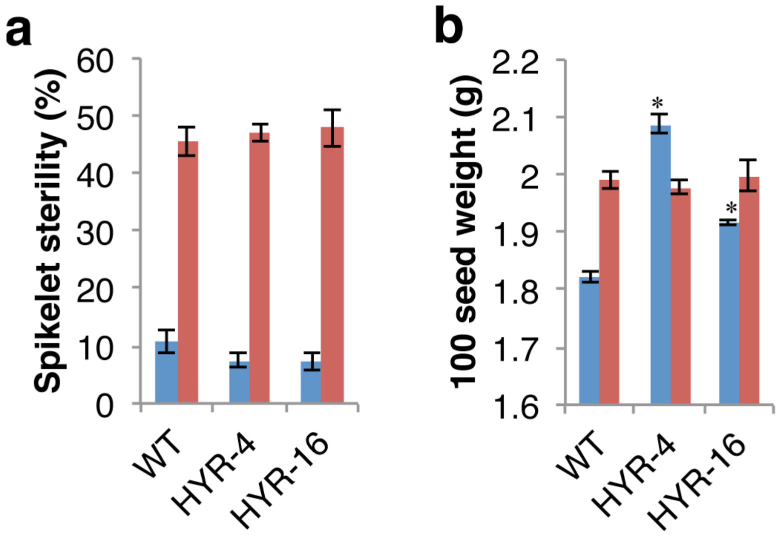

Supplementary Figure 8. Effect of high temperature on HYR lines**.** (**a**) Spikelet sterility of HYR lines (red bars) and wild-type (WT) (blue bars) under normal control and high temperature stress. (**b**) The grain weight measured as 100 seed weight for HYR and WT under control temperature and high day/night temperatures. Plants at early boot stage were exposed to high day/night temperature of 36/26 ^{o}C until physiological maturity. Values are the mean ± s.e.m. (n>6) and '*' indicates significant difference from wild-type, t-test at P<0.05.

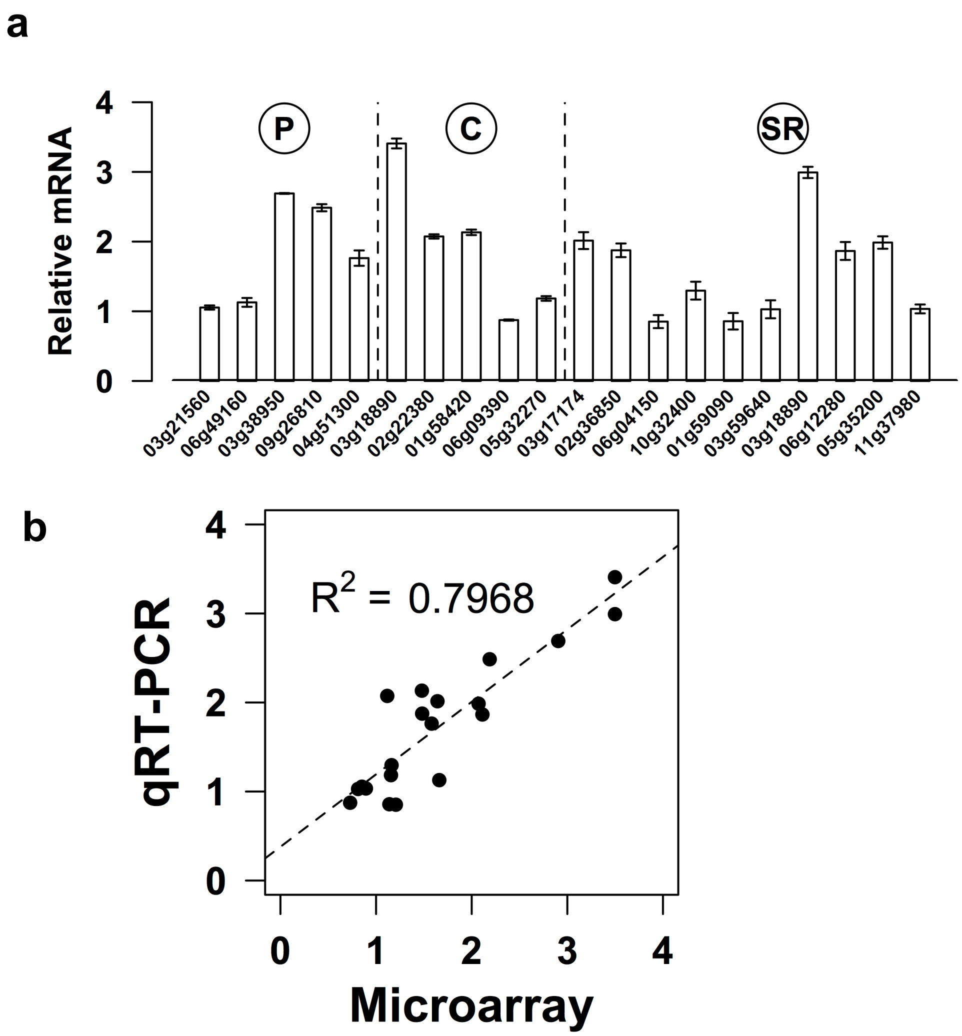

Supplementary Figure 9. Gene expression validation of microarray data by qRT-PCR.

(**a**) Relative gene expression (LogRatio) measured by qRT-PCR showing mean ± s.e.m., of a set of genes from the 'P' (photosynthesis) and 'C' (carbon metabolism) gene sets identified from HYR microarray data (see Methods). In addition, for reference, plotted are the differential expression levels of known stress-related (SR) genes. (**b**) The scatter plot shows the level of differential expression of genes in (c) as recorded by the microarray (x-axis) and qRT-PCR (y-axis).

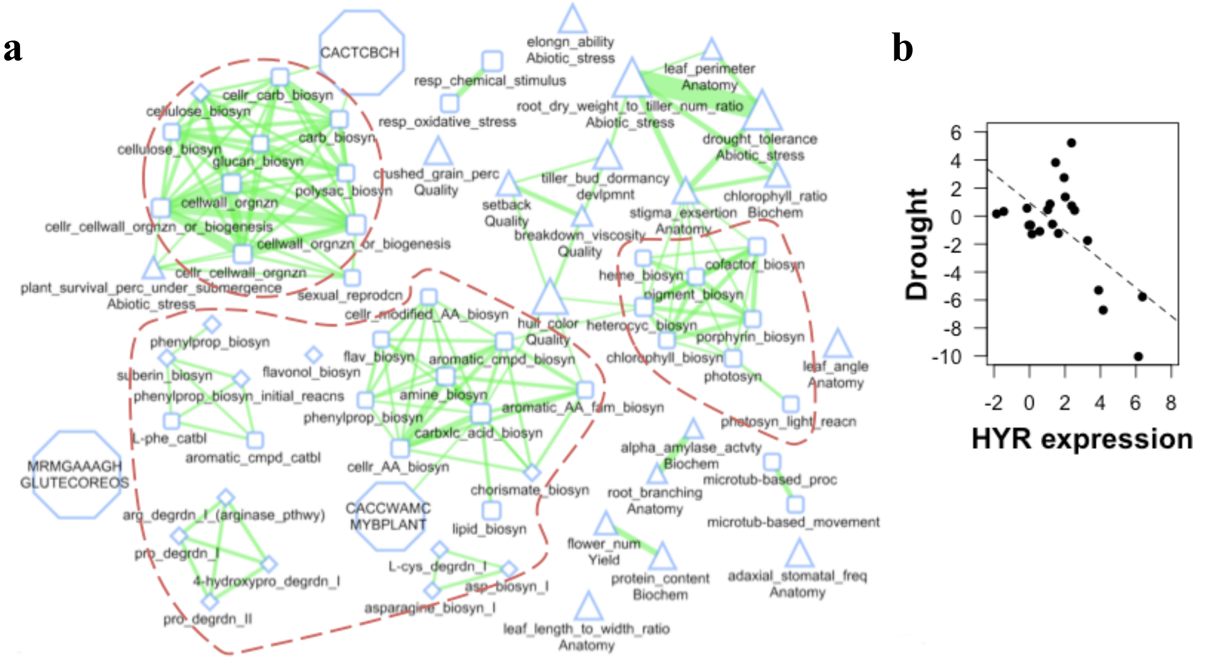

Supplementary Figure 10. HYR regulates expression of rice genes in PCM and other biological processes. (**a**) Gene set enrichment analysis led to the identification of several biological processes, metabolic pathways and quantitative traits among the genes up-regulated upon HYR expression. Cis-regulatory element (CRE) discovery suggested DNA motifs that are strongly associated with the HYR-induced genes, potentially regulating the enriched biological processes. Nodes in the graph, depending on shape, correspond to subsets of HYR-induced genes annotated with enriched processes (rounded rectangle), pathways (diamond) or traits (triangle), or containing associated CREs (octagon). Edges represent significant overlap ($q < 0.1$) between the subsets. Dashed curves circumscribe groups of subsets related to ‘carbohydrate metabolism’ (C), ‘photosynthesis’ (P) and ‘amino acid metabolism’. (**b**) The scatter plot shows the association scores of the ‘C’ and ‘P’ gene sets with global differential expression upon HYR expression (x-axis) and drought stress (y-axis). The dashed line corresponds to the regression line.

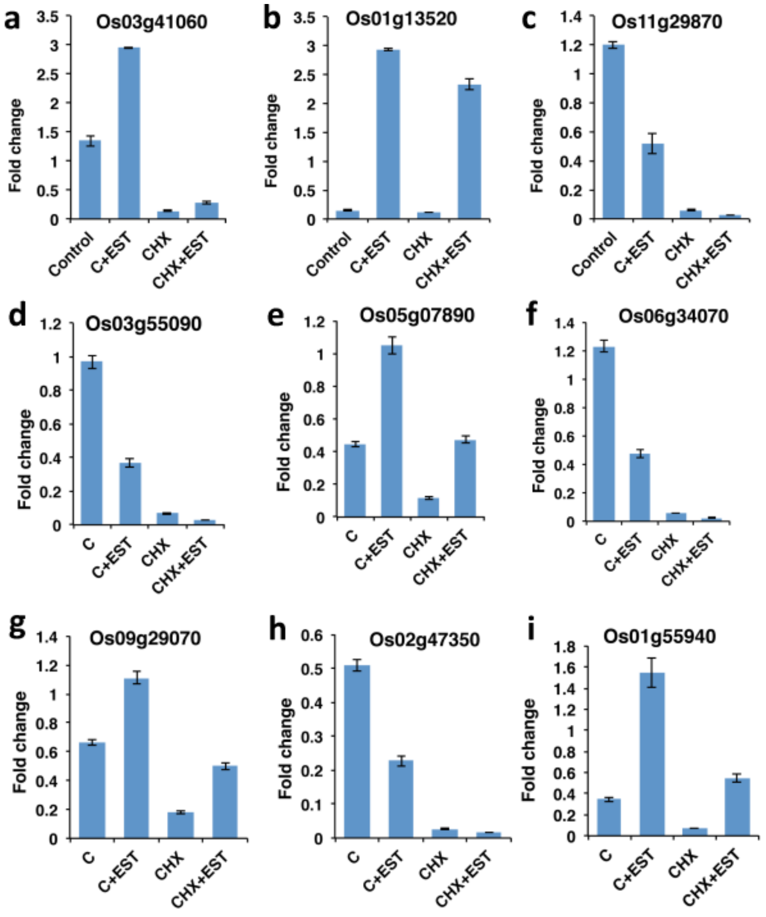

Supplementary Figure 11. HYR and its downstream TFs directly regulate transcription of PCM genes. Gene fusions of HYR and other TFs with the receptor (HER) domain were tested for transactivation of target genes in the genome (shown in **Fig. 6**) by RT-PCR using gene specific primers (**Supplementary Table 7**, see Online Methods for details), with data shown as mean ± s.e.m. Protoplasts transformed with HER gene fusions of HYR, GASR2 and ARF1, were tested in the 4 treatments and protoplasts used for RNA isolation and RT-PCR analysis: Treatments C -control Nipponbare; C+EST -estradiol control; CHX –cycloheximide (CHX) treatment; CHX+EST –CHX and EST treatment. (**a-c**) HYR-HER expression for transactivation of downstream TFs GASR2, ARF1 and WRKY72; (**d-f**) TF GASR2-HER expression to test expression of GASR2 downstream genes; (h-i) ARF1-HER expression to test expression of ARF1 regulated genes.

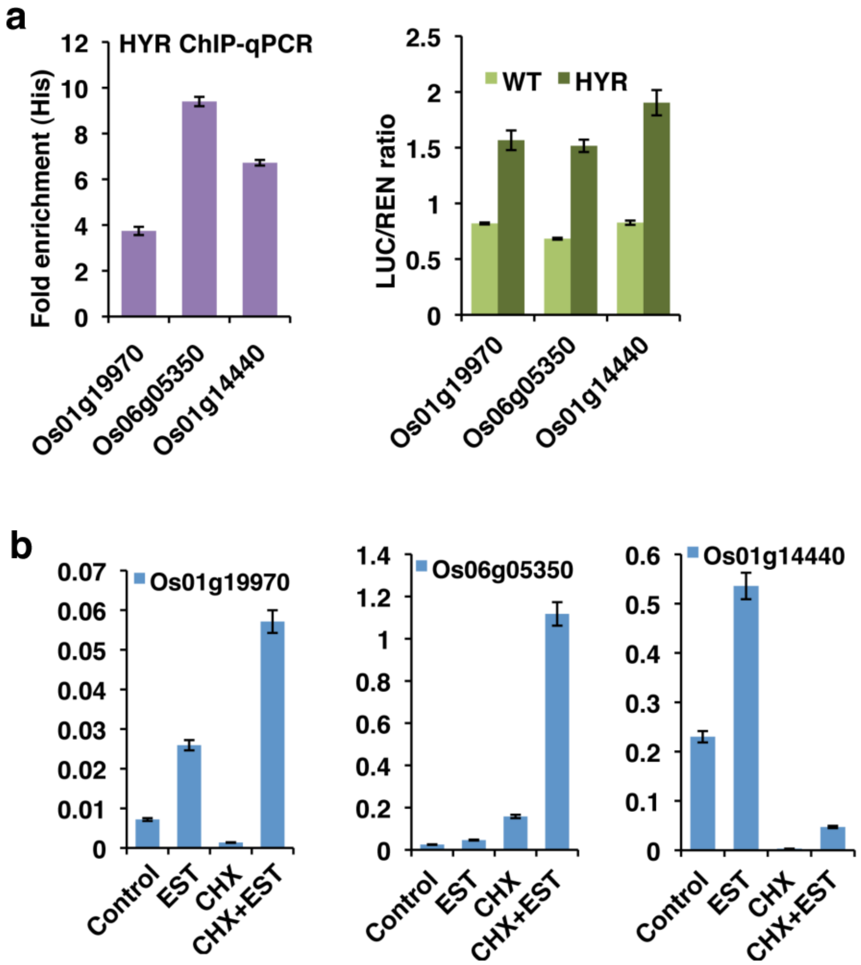

Supplementary Figure 12. HYR transcriptionally regulates the expression of drought responsive genes. Microarray data of HYR expressing genotype was used to identify TF gene that were up-regulated by HYR and known to be up-regulated under drought (**Fig. 1a**), data represents mean ± s.e.m. (**a**) The HYR/drought regulated TFs, Os01g19970 (MYB), Os06g05350 (Whirly), and Os01g14440 (OsWRKY1) were tested by ChIP-qPCR for binding of HYR to their promoters. HYR activates the transcription of the drought-responsive TFs in luciferase transactivation experiments. (**b**) HYR directly regulates expression of the drought-responsive genes using HYR-HER fusions in EST/CHX assays.

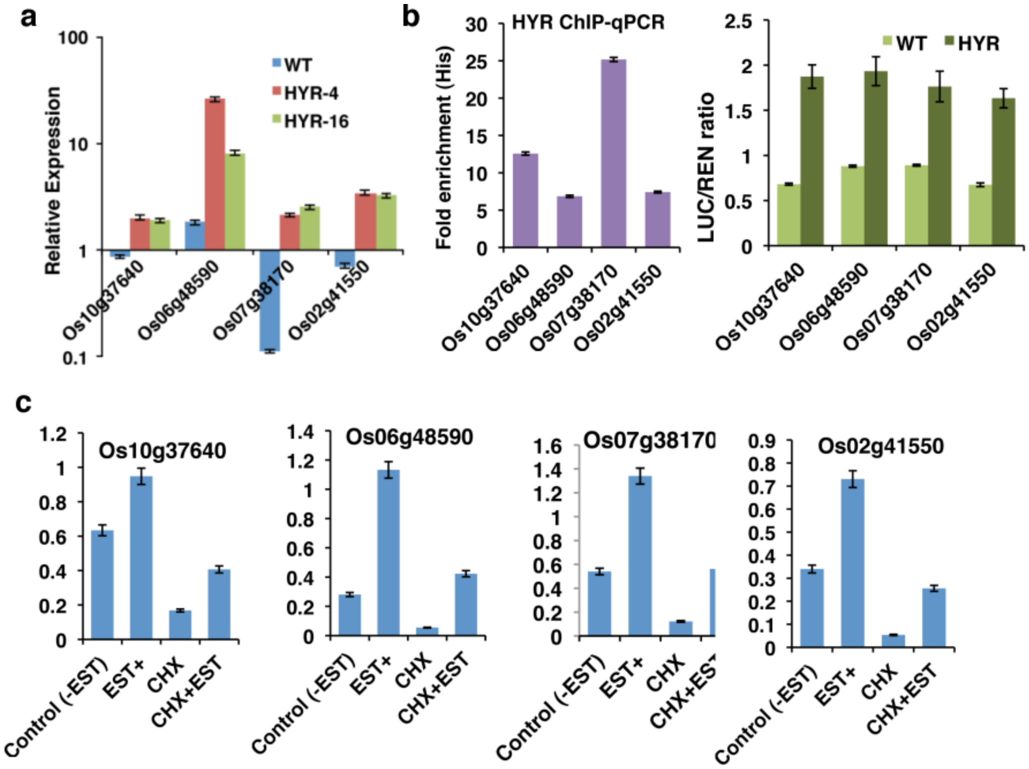

Supplementary Figure 13. HYR directly regulates the expression of heat-responsive genes.

The heat responsive genes were identified from published literature as described in Methods, and that were differentially expressed in HYR lines, data representing mean ± s.e.m. (a) qPCR analysis of the heat-responsive genes in HYR lines showing induction. (**b**) ChIP-qPCR analysis shows HYR binds to the heat-responsive promoters *in vivo* and activates the promoter in luciferase transactivation assays. (**c**) HYR directly regulates expression of the heat-responsive genes using HYR-HER fusions in EST/CHX assays.

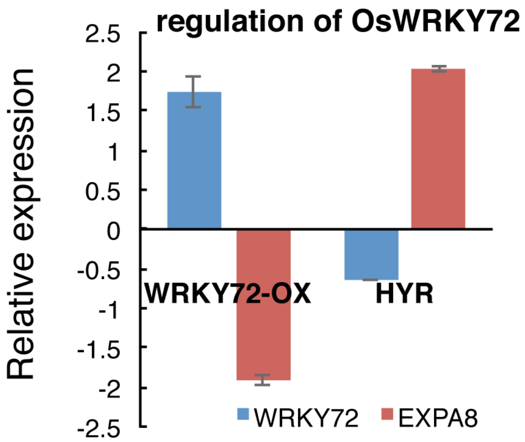

Supplementary Figure 14. Regulation of root growth by WRKY72

Rice protoplasts were cotransformed with i) 35S:WRKY72 alone (WRKY72-OX), ii) 35S:HYR, while non-transfected rice protoplasts served as control and incubated for 24h at 28^{0}C. Total RNA was isolated from the rice protoplast and qPCR analysis of WRKY72 and EXPA8 were done compared to control. Values are the mean ± s.e.m.of three biological replicates.

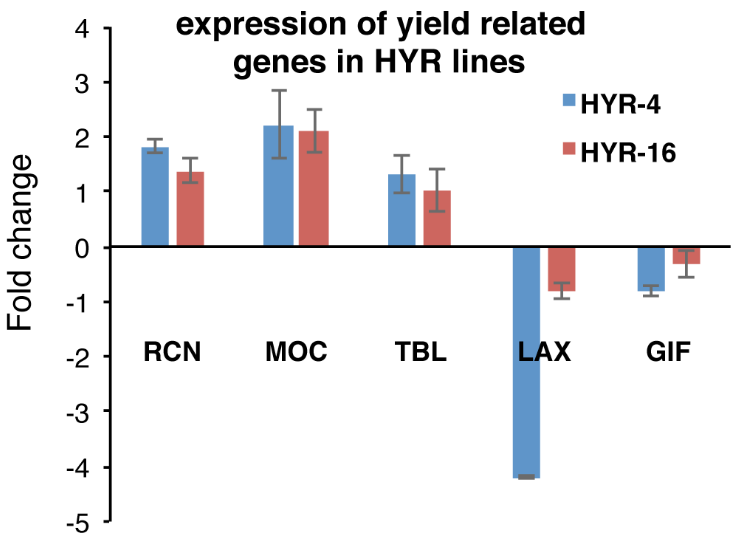

Supplementary Fig 15. Expression analysis of yield related genes in HYR genotypes.

To study the expression of genes related to tillering in HYR genotypes qPCR was done with two HYR lines (HYR-4 and HYR-16). The genes tested for qPCR are: RCN, MOC, TBL, LAX, GIF (Tripathi et al., 2012). The results presented here are expressed as fold change relative to the WT (Nipponbare) plants grown under the same greenhouse conditions and are mean ± s.e.m. of three biological replicates.

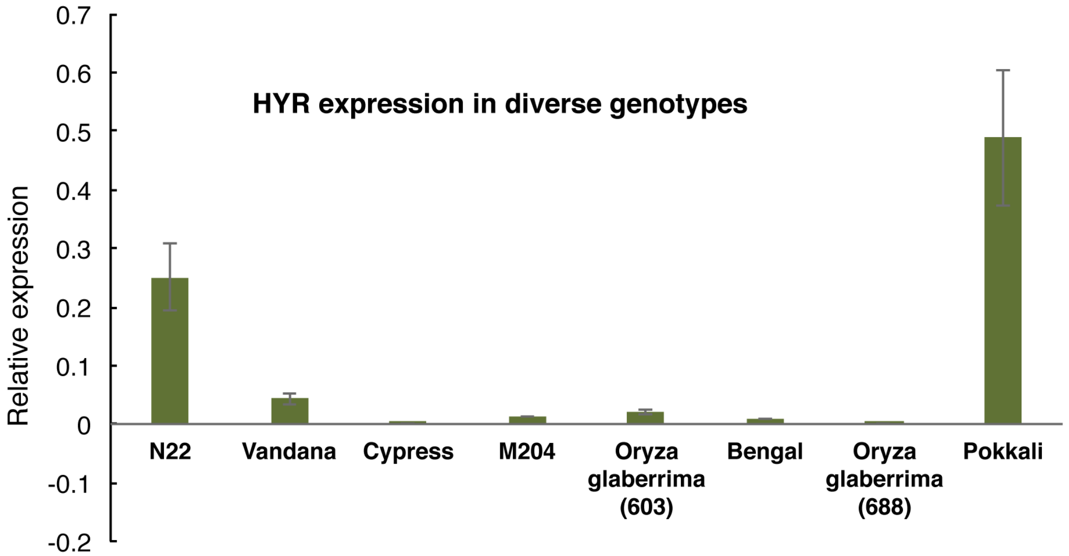

Supplementary Fig 16. Expression analysis of HYR in diverse rice genotypes

The expression of HYR was studied by qPCR using total RNA isolated from 25-day old leaf tissue of rice genotypes grown under control conditions. The results represented here are intrinsic values and are mean ± s.e.m. of three biological replicates.

Supplementary Table 1 Differential expression of photosystem II and electron transport genes in HYR lines as classified by MapMan. The table shows gene names, expression values and putative functions.

Gene	Log ratio	Q.value	Function
**PSII**			
LOC_Os01g59090	1.14	0.038	thylakoid lumenal 20 kDa protein, putative, expressed
LOC_Os02g36850	1.482	0.039	oxygen evolving enhancer protein 3, identical, putative, expressed
LOC_Os03g17174	1.64	0.02	PsbP, putative, expressed
LOC_Os03g21560	0.852	0.035	photosystem II 11 kD protein, putative, expressed
LOC_Os03g53640	1.741	0.021	photosystem II 11 kDa protein
LOC_Os04g44200	0.999	0.044	oxygen-evolving enhancer protein 3, chloroplast precursor, putative
LOC_Os08g25900	1.153	0.027	PsbP, putative, expressed
LOC_Os08g39430	1.579	0.039	thylakoid lumenal 19 kDa protein, chloroplast precursor, putative
LOC_Os10g21310	2.019	0.025	photosystem II P680 chlorophyll A apoprotein, putative, expressed
LOC_Os06g49160	1.661	0.017	thylakoid lumenal 16.5 kDa protein, chloroplast precursor, putative, expressed
LOC_Os10g32400	1.162	0.027	PsbP, putative, expressed
LOC_Os12g29570	0.9	0.048	Chlorophyll a-b binding protein
**Electron Transport**			
LOC_Os04g33630	2.365	0.011	2Fe-2S iron-sulfur cluster binding domain containing protein, expressed
LOC_Os07g38000	3.042	0.011	cytochrome c, putative, expressed
LOC_Os08g35710	2.601	0.03	expressed protein
LOC_Os02g22260	0.908	0.028	fruit protein PKIWI502, putative, expressed
LOC_Os01g03050	-0.712	0.043	fruit protein PKIWI502, putative, expressed
LOC_Os01g64120	-3.921	0.02	2Fe-2S iron-sulfur cluster binding domain containing protein, expressed
LOC_Os03g11450	-0.698	0.044	expressed protein
**LHC1 and PS1**			
LOC_Os10g39150	1.593	0.023	photosystem I P subunit
**LHC2**			
LOC_Os01g52240	0.822	0.03	chlorophyll A-B binding protein, putative, expressed
LOC_Os02g52650	1.379	0.025	chlorophyll A-B binding protein, putative, expressed
LOC_Os07g38960	1.309	0.049	chlorophyll A-B binding protein, putative, expressed
LOC_Os09g26810	2.188	0.012	chlorophyll A-B binding protein, putative, expressed

Supplementary Table 2: Rice Microarray Datasets

	Dataset	Num Samples	Num Groups	Biological context	Genotype	Description
Affymetrix - ArrayExpress	E-MEXP-2267	36	12	Environmental condition response	japonica cv. Amaroo	Transcription profiling time course of rice germination under anaerobic conditions, aerobic to anaerobic switch and anaerobic to aerobic switch
	E-MEXP-1766	15	5	Environmental condition response	japonica cv. Amaroo	Transcript abundance profiles were examined over the first 24 hours of germination in rice grown under aerobic conditions
Affymetrix - GEO	GSE14403	23	8	Environmental condition response	indica cv. FL478, Pokkali, IR63731 and IR29	Root-specific transcriptional profiling of contrasting rice genotypes in response to salinity stress
	GSE4471	12	4	Environmental condition response	japonica cv. Azucena and indica cv. Bala	Expression data from rice varieties Azucena and Bala grown in 0 and 1ppm arsenate
	GSE6901	12	4	Environmental condition response	indica cv. IR64	Expression data for stress treatment in rice seedlings
	GSE10054	6	3	Environmental condition response	indica cv. Zhenshan 97	Expression information of splicing factor OsSKIPa knock-down and overexpressed rice
	GSE15448	9	3	Environmental condition response	indica cv. IR64	Glycinebetaine-induced water-stress tolerance in codA-expressing transgenic indica rice
	GSE11175	6	2	Environmental condition response	japonica cv. Zhonghua 11	Comparison of transcriptome profile between wild-type and dst mutant plants
	GSE14275	6	2	Environmental condition response	NA	Expression data for heat shock in rice seedlings
	GSE6908	4	2	Environmental condition response	japonica cv. Nipponbare	Transcript Profiling of the Aerobic and Anoxic Rice Coleoptile
	GSE6893	45	15	Tissue/ Developmental stage	indica cv. IR64	Expression data for reproductive development in rice
	GSE11966	10	5	Tissue/ Developmental stage	japonica cv. Zhonghua 11	Expression data from rice embryo, endosperm, root, leaf and seedling
	GSE16265	10	2	Tissue/ Developmental stage	japonica cv. Nipponbare and indica cv. 93-11	SNEP: Simultaneous detection of nucleotide and expression polymorphisms using Affymetrix GeneChip
	GSE17194	4	2	Tissue/ Developmental stage	indica cv. Zhongxian 3037	Genome-wide gene expression profiling of rice Indica cultivar Zhongxian 3037 and mutant phoenix (pho) panicle
	GSE9498	6	2	Tissue/ Developmental stage	japonica cv. Zhonghua 11	Global gene expression profiles of Oryza sativa wild type Zhonghua11 and mutant gif1 in filling stage
BGI-Yale - GEO	GSE13131	168	42	Tissue/ Developmental stage	japonica cv. Nipponbare	A transcriptome atlas of rice cell types uncovers cellular, functional and developmental hierarchies

Supplementary Table 3 List of Primers used for Chip-qPCR

Os03g55090 F	TGTTAAGAAATAATAATAAGATCT
Os03g55090 R	ACTCCAAGGTTTATATTGTCA
Os06g34070 F	AGAAACAGGAACATACCAGTA
Os06g34070 R	CTGGTGCGCCAGGAGGAG
Os05g07890 F	TTGGATAGCTAGATGACATAATTT
Os05g07890 R	GATCTATGGTTCGAATTATTT
Os09g29070 F	GTGACCAAATTAATATTGCCC
Os09g29070 R	AGAATGTAGGAAGACAAGGTC
Os02g47350 F	TATTGGCAGATGTCTTTTAATACA
Os02g47350 R	CAAGTTTAGGGACCGGTGATG
Os01g55940 F	TGTCCTTTATATTATAGGTTGAT
Os01g55940 R	GAGTCTGATCTTTTGGCCACA
Os07g36560 F	GCAACCCGCAGTAGGCTAACA
Os07g36560 R	CCTTGCTGACGTTGAATCATC
Os10g37640 F	TCTCTCTCTCTCTCTCTCTCC
Os10g37640 R	ACATGAGAGCGGTTGGTGAAG
Os06g48590 F	ATGCACCATGCAGCACTAAGT
Os06g48590 R	AGCTAGTCAGATATCGAAACGTAC
Os07g38170 F	AAAGGGGCCCTAAACCTAG
Os07g38170 R	TTTAATCCCATCTAAACAGGC
Os02g41550 F	CCACGCGGAAATCGGGGC
Os02g41550 R	CCTCACAGGCGACACGGG
Os02g36850 F	CGGCCCGCCCCAACAAAG
Os02g36850 R	TCGACCCCCTCGTAGCCT
Os03g21560 F	GGCCAATCCCGTCTCCTTT
Os03g21560 R	CGTCGGCTTATCCCCTTA
Os08g39430 F	GGAGGGAGACGGGGTGGG
Os08g39430 R	TCAGAAAGGGAGGAGAGCCGT
Os04g33630 F	GTGGGTGGCGGTGGCAAG
Os04g33630 R	CACGACGGGCCTCAGCTC
Os02g52650 F	GGCGCGGTCGAACGAGCT
Os02g52650 R	GTTTAGCCATTAGTCAAAGCAATC
Os07g38960 F	CCAGTGATGTGACCATGGGTA
Os07g38960 R	AGGACTCCGAGATCTTTCGAT
Os03g41060 F	ATGCTACTACTCCTCCAAATG

Os03g41060 R	GCACTTCACACCGTACCG
Os01g19970 F	CTCCCACGTGTCACCCCCA
Os01g19970 R	TGCTTTTCCTCCCCGGGC
Os01g13520 F	AGCACCAAAGAAGGGGAGGAC
Os01g13520 R	CTCTGCTCTCTCTCGTGTCAG
Os01g14440 F	GACTCTCCCACTCTCTTCTC
Os01g14440 R	TGGTCGACCTGTGGAACG
Os06g05350 F	TAAGGCCATAATCTATTTAGG
Os06g05350 R	TCAAGCGATCAGGAATTCAGG
Os11g29870 F	GAACCTAGCTAGCTAGCTCT
Os11g29870 R	AGAGCTAGCTAGCTAGGTTC

Supplementary Table 4 List of Primers used for Luciferase Transactivation Assay

Os10g37640 F	ACGTTCTAGATCTCTCTCTCTCTCTCTCTCC
Os10g37640 R	ACGTGGATCCAATTTAGTGGAGTACTACTCCGTT
Os06g48590 F	ACGTTCTAGAATGCACCATGCAGCACTAAGT
Os06g48590 R	ACGTGGATCCGCGCCTCTTGGCTTCTTCCTC
Os07g38170 F	ACGTTCTAGACCGGACAACCTTAAGTTATTG
Os07g38170 R	ACGTGGATCCTTTAATCCCATCTAAACAGGC
Os02g41550 F	ACGTTCTAGACCACGCGGAAATCGGGGC
Os02g41550 R	ACGTGGATCCGCGACCCAGGCTGTAACCAAA
Os03g55090 F	ACGTTCTAGATGTTAAGAAATAATAATAAGATCT
Os03g55090 R	ACGTGGATCCTAGGCCGGCGGCCCGAAG
Os06g34070 F	ACGTTCTAGACCTTGAGGAGCTAGCCAAAAG
Os06g34070 R	ACGTGGATCCCTGGTGCGCCAGGAGGAG
Os05g07890 F	ACGTTCTAGACTAAATTTCGAGTCCCATCGG
Os05g07890 R	ACGTGGATCCGATCTATGGTTCGAATTATTT
Os09g29070 F	ACGTTCTAGAGAGCGACGGCGGGGGGCG
Os09g29070 R	ACGTGGATCCAAAAAGAAGTGGGCCCATCTAGTA
Os02g47350 F	ACGTAAGCTTTATTGGCAGATGTCTTTTAATACA
Os02g47350 R	ACGTGGATCCTACTAACTCCATATATCTAGATCT
Os01g55940 F	ACGTTCTAGATGTCCTTTATATTATAGGTTGAT
Os01g55940 R	ACGTGGATCCACACGGCTCTAACTAAAAATCCAT
Os07g36560 F	ACGTAAGCTTAACCGCAAGGCTAGGCTTTTG
Os07g36560 R	ACGTGGATCCCCTTGCTGACGTTGAATCATC
Os01g19970 F	ACGTTCTAGACTTATGCACGAGACAAGGATA
Os01g19970 R	ACGTGGATCCTGCTTTTCCTCCCCGGGC
Os01g14440 F	ACGTTCTAGAAAAGAACAATTCTACCTCCCA
Os01g14441 R	ACGTGGATCCTGGTCGACCTGTGGAACG
Os06g05350 F	ACGTTCTAGAAGGTTTCTTAATTTGTTTTCTTGA
Os06g05350 R	ACGTGGATCCCGCCGGCCTGTGGCTAGG
Os02g36850 F	ACGTTCTAGACTAGGGGAGCTTTAGATTTTAAGA
Os02g36850 R	ACGTGGATCCTCGACCCCCTCGTAGCCT
Os03g21560 F	ACGTAAGCTTTCCTTTTACTTGATTTTCTGCTG
Os03g21560 R	ACGTGGATCCCGTCGGCTTATCCCCTTA
Os08g39430 F	ACGTTCTAGAACCATTTCTATGAACACCTCCA
Os08g39430 R	ACGTGGATCCTCAGAAAGGGAGGAGAGCCGT

Os04g33630 F	ACGTTCTAGATCGGTGGTTTAGCTGGCGG
Os04g33630 R	ACGTGGATCCCACGACGGGCCTCAGCTC
Os02g52650 F	ACGTTCTAGAAAGAGTATATTAAATACCGGTGTT
Os02g52650 R	ACGTAGATCTGTTTAGCCATTAGTCAAAGCAATC
Os07g38960 F	ACGTTCTAGAAATGGTAAGTGTAGATTTTTGATG
Os07g38960 R	ACGTGGATCCAGGACTCCGAGATCTTTCGAT
Os03g41060 F	GATCTCTAGATCGATGGGAAATATGGGAGCC
Os03g41060 R	GATCGGATCCATCGGTTACCTGAAGCAGCGT
Os01g13520 F	GATCTCTAGAGCCAAATGTTATTCTATCTATTAC
Os01g13520 R	GATCGGATCCAGAGAAGAAGAAGAAGAAGAG
Os11g29870 F	GATCTCTAGAGATTATAAGACGATATGACAGGTG
Os11g29870 R	GATCGGATCCGAAGTTCTCCATCTCGACCAA
luc f	GATCGGATCCATGGAAGATGCCAAAAACATTAAG
luc r	GATCGGTACCTTAGACGTTGATCCTGGCGCT
hlucf	GATCGGATCCATGGCTTCCAAGGTGTACGAC
hluc r	GATCGGTACCTTAGACGTTGATCCTGGCGCT
HYR F	GATCGGATCCATGGATCGAGACGAGAGCTTG
HYR R	GATCGGTACCTCAGGAATGGTTCCACAGGCT
Os11g29870 F	ACGTGGATCCATGGAGAACTTCCCGATACTC
Os11g29870 R	ACGTGGTACCCTACTGGAACATGTGGGAAGC
Os03g41060 F	ACGTGGATCCATGAAGACCCGCCGGGCC
Os03g41060 R	ACGTGGTACCTCAGGGGCACTTGGGCCT
Os01g13520 F	ACGTGGATCCATGTCGTCGCAAGGAGCAGGA
Os01g13520 R	ACGTGGTACCCTAAAACCCAGTCTTCTGATCCTG

Supplementary Table 5 Primers used for identification of Direct targets by HER qPCR analysis

Os02g36850 F	CCATGTCGTGGAAGTACGTG
Os02g36850 R	TCGGTCATGTTATCGACGAG
Os03g21560 F	GTCGACGATCAACATGGACA
Os03g21560 R	TACATCTCCCTGAAGGACGG
Os08g39430 F	TCAAGGTGTACTACGGCACG
Os08g39430 R	GGTTGAAGAACTCGCTGTCC
Os04g33630 F	ACCGTCACCACACCAATTCT
Os04g33630 R	CGTCCAGGATGTAGGTGTCC
Os02g52650 F	GGCACTCTTCTTTGTCCAGC
Os02g52650 R	TAGCCAACCTCCCATTCTTG
Os07g38960 F	AAGATCGGCATCCTCAACAC
Os07g38960 R	CAGCTCCTTGATCTTCTCCG
Os07g36560 F	GGAAGGATCAGTGAGGTGGA
Os07g36560 R	GCGTCACCTGAATGAAGAGG
Os09g29070 F	CCGAGAATCCATCTCCGTTA
Os09g29070 R	CTTCACCTGCTGCCTTTTTC
Os02g47350 F	AGGAGGTCAAGTGGATGGTG
Os02g47350 R	AAACCCGGTGTCTCTGTGTC
Os01g55940 F	CTCTACAGCCTCCTCATGCC
Os01g55940 R	ACACGTTGTAGGGGTCGAAG
Os03g55090 F	GTTTGTGCCTGACCCAAGAT
Os03g55090 R	GTACTCGTGAATCGTCCGGT
Os05g07890 F	GTACGGCGTCTGCTACCTCT
Os05g07890 R	GAGCCGAAGTAGAAGGTGGA
Os06g34070 F	AGCCCGAAAAGTTTGGATTT
Os06g34070 R	ATACCATCCCAGCTCCAGTG
Os10g37640 F	GCTCAAGGCAGAGAAATTGG
Os10g37640 R	GGGCATCGTTCTTTCTACCA
Os06g48590 F	GGCTCGCACAAACAACACTA
Os06g48590 R	TCCTGGAAATATTGGCTTGC
Os07g38170 F	AACGCCTGGCTCAAGAAGTA
Os07g38170 R	AGAAGAAGGAGCGCTTGGAT
Os02g41550 F	TGCACCGAGCTGTATTCTTG
Os02g41550 R	CCTCCACAACCTCACCATCT

Os03g41060 F	CGCTGCTAATGCTTCTCCTC
Os03g41060 R	ACTTGGGCCTCTTCCTGG
Os06g05350 F	TTTCAAGGGGAGGAGTGATG
Os06g05350 R	CCTAGTGTACGCCCGTGAAT
Os01g13520 F	TCTGCAGTGGTCAGGTTCTG
Os01g13520 R	TCTTCTCATGCTGTTGTGGC
Os01g19970 F	GGTCTCACATCAGATGGGCT
Os01g19970 R	CCACTCTCTGCAGAAGACCC
Os01g14440 F	AGGACCAAGAACGTGGTGAC
Os01g14440 R	CTTCCTCATGTTGGCATCCT
Os11g29870 F	GGCCGTCAAGAACAACAAAT
Os11g29870 R	TGAGGATGTGCTCGAAGTTG
